# Exceptional Visual‐Opsin Coexpression and Phenotypic Diversity in Outer‐Retinal Photoreceptors of Caenophidian Snakes

**DOI:** 10.1002/cne.70092

**Published:** 2025-10-07

**Authors:** Einat Hauzman, Silke Haverkamp, Juliana H. Tashiro, Irene L. Gügel, Natalia F. Torello‐Viera, Thaís B. Guedes, Pavel Němec, Nicholas R. Casewell, Cassandra M. Modahl, Maria Ermelinda Oliveira, Ana Lúcia C. Prudente, Daniel O. Mesquita, Dora Fix Ventura, David J. Gower

**Affiliations:** ^1^ Department of Experimental Psychology, Psychology Institute University of São Paulo São Paulo Brazil; ^2^ Natural History Museum London UK; ^3^ Department of Computational Neuroethology Max Planck Institute for Neurobiology of Behavior Bonn Germany; ^4^ Institute of Biosciences Federal University of Mato Grosso Cuiabá Brazil; ^5^ Department of Biodiversity, Bioscience Institute São Paulo State University Julio de Mesquita Filho UNESP Rio Claro Brazil; ^6^ Department of Zoology, Faculty of Science Charles University Prague Czech Republic; ^7^ Centre for Snakebite Research & Interventions, Liverpool School of Tropical Medicine Liverpool UK; ^8^ Institute of Biological Sciences Federal University of Amazonas Manaus Brazil; ^9^ Museu Paraense Emílio Goeldi (MPEG) Belém Pará Brazil; ^10^ Department of Systematic and Ecology Federal University of Paraíba João Pessoa Paraíba Brazil

**Keywords:** Multiopsin cones, opsin expression, photoreceptor morphology, retinal topography, snake retina

## Abstract

Snakes are a valuable yet understudied taxon for investigating evolutionary adaptations in the vertebrate retina. They possess up to three visual pigments: a short‐wavelength‐sensitive opsin (SWS1), a medium/long‐wavelength‐sensitive opsin (LWS), and rhodopsin (RH1). Nocturnal snakes have duplex retinas containing both rod and cone photoreceptors, whereas diurnal caenophidian (“advanced”) snakes exhibit simplex “all‐cone” retinas, lacking morphologically typical rods. In this study, we analyzed photoreceptor morphology in the retinas of caenophidian snakes using high‐resolution scanning electron microscopy (SEM) and examined visual‐opsin expression patterns with immunohistochemistry (IHC). Our analyses revealed remarkable interspecific variability in visual‐cell morphology. Light microscopy showed that in all sampled diurnal caenophidians, photoreceptors expressing RH1 exhibit a gross cone‐like morphology. However, SEM analysis revealed a subset of photoreceptors with distinct features—thinner inner segments and rod‐like synaptic terminals—suggesting they are transmuted, cone‐like rods. In retinal sections from nocturnal caenophidian snakes, coexpression of the cone opsins SWS1 and LWS in individual cones was observed, whereas rhodopsin expression remained restricted to morphologically typical rods and showed no coexpression. In contrast, diurnal caenophidians commonly coexpress rhodopsin and SWS1 in single cones, with some instances of triple coexpression (SWS1, RH1, and LWS) in single cones. We evaluated the patterns of spatial distribution of RH1‐ and SWS1‐expressing photoreceptors, as well as SWS1 + RH1 multiopsin cones, in wholemounted retinas of ten species. Our findings revealed considerable species‐specific variation in photoreceptor density, topography, and opsin coexpression patterns. IHC results suggest that in some species, rhodopsin is not only expressed in transmuted, cone‐like rods but may also be co‐opted by UV/violet‐sensitive (SWS1‐expressing) cones. These findings underscore the exceptional diversity and adaptive innovation in snake visual systems. The unique features and striking interspecific differences in their photoreceptors highlight snakes as an outstanding taxon for studying vertebrate visual‐system function and evolution.

## Graphical abstract

In snakes, profound differences in retinal architecture are observed between diurnal and nocturnal species. Additionally, in the rod‐dominated retinas of nocturnal snakes, coexpression of the cone opsins SWS1 and LWS in individual cones is common, while rhodopsin (RH1) is restricted to typical rods. In the cone‐dominated retinas of diurnal species, rhodopsin is expressed in transmuted, cone‐like rods and frequently coexpressed with SWS1 in some single cones, with some instances of triple coexpression (SWS1, RH1, and LWS) in single cones.

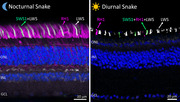

## 1 Introduction

Two main types of photoreceptors in the outer part of the retinas of vertebrates, rods and cones, typically operate in contrasting light intensities (Lamb 2009). These photosensitive neurons absorb photons and convey luminous information to inner‐retinal neurons, initiating visual processing. Rods are highly sensitive, capable of generating a physiological response upon the absorption of a single photon (Rieke and Baylor 1998). Typically having long, subcylindrical outer segments that contain large amounts of rhodopsin photopigment (RH1), rods are responsible for vision under scotopic (low‐light) conditions. Cones, in contrast, typically have smaller, tapered outer segments, are less sensitive, and generate a faster response, and are therefore chiefly responsible for photopic (daylight) vision. Different classes of visual opsins expressed in subtypes of cones form different photopigments that vary in their peak spectral absorption, allowing sensitivity from short (ultraviolet [UV] to violet) to long (red) wavelengths (Lamb 2013). Cone subtypes are feature channels that feed into downstream parallel pathways for decoding both spectral and nonspectral aspects of vision (Baden 2024).

In squamate reptiles (snakes and lizards), the established rod and cone dichotomy of the vertebrate duplex retina is challenged by unusual occurrences of (at least superficially) simplex retinas, seemingly having only rods or cones as determined by gross morphology. Based on broad comparative analyses of the visual cells of squamates, the visual anatomist Walls (1942) suggested that cones and rods are not immutable units but can interconvert evolutionarily via transitional states as adaptations to changes in species’ daily activity patterns. Walls coined the term “transmutation” to describe the morphological (and presumably physiological) evolutionary transformation of photoreceptors from cones to transmuted, rod‐like cones and from rods to transmuted, cone‐like rods. Support for Walls’ transmutation theory has more recently been found from molecular genetic data, especially for the expression of RH1 and the typical cone opsins SWS1 and LWS in caenophidian snakes with simplex (seemingly all‐cone or all‐rod) retinas, and for the RH1 that is expressed in transmuted, cone‐like rods having cone opsin‐like functional characteristics (Bhattacharyya et al. 2017; Schott et al. 2016; Simões et al. 2016).

In snakes, the absence of some ocular structures (e.g., ciliary muscles, eyelids), visual‐cell types (blue and green cones, typical tetrapod double cones), and two cone visual opsins (short‐wavelength‐sensitive opsin [SWS2] and RH2), all present in lizards, points to visual‐system simplification during the origin of snakes from lizards, presumably as adaptations to burrowing habits and/or nocturnal or crepuscular activity (Gower et al. 2022). Snakes from the relatively “basal” and paraphyletic Henophidia group (including pythons, boas, and pipesnakes) have duplex retinas dominated by typical rods (with RH1) along with two types of single cones, sensitive to short (UV) and to long wavelengths with the SWS1 and LWS visual opsins, respectively. Under Baden et al.’s (2025) newly proposed nomenclature for rod and cone subtypes, these rods are termed PR0, and the LWS and SWS1 single cones are PR1 and PR4. This duplex retinal pattern and photoreceptor complement likely approximates that of the ancestral snake (e.g., Gower et al. 2022).

In the “advanced” and monophyletic Caenophidia (including vipers, cobras, and colubrids), high species richness is accompanied by great diversity of retinal patterns and visual‐cell morphologies. Parallel transitions between diurnal and nocturnal habits in many lineages apparently led to new evolutionary trajectories of the visual system. Nocturnal caenophidian species from different families have duplex retinas, with a prevalence of typical rods with RH1 (PR0), in addition to three types of cones: large single LWS cones (PR1), small single SWS1 cones (PR4), and one type of double LWS cone absent in non‐caenophidian snakes and morphologically distinct from the double cones (PR5 + PR6) of other tetrapods (Underwood 1968). On the other hand, many diurnal caenophidian species lack (morphologically) typical rods and have so‐called “all‐cone” retinas (Underwood 1967; Walls 1934, 1942; Wong 1989), with four cone‐like photoreceptors: large single (red/green, PR1) and double LWS cones (red/green; resembling the double cones of nocturnal caenophidian snakes), small single SWS1 cones (UV/violet; PR4), and a second type of small single cone‐like photoreceptors, sensitive to middle wavelengths (Hart et al. 2012; Sillman et al. 1997). Using immunohistochemistry (IHC), Schott et al. (2016) showed the expression of rhodopsin and rod transducin in a subpopulation of small cone‐like photoreceptors of a diurnal colubrid, revealing the maintenance of the rod phototransduction machinery. Transmission electron microscopy (TEM) revealed that one subset of small photoreceptors has less‐tapered outer segments and less‐bulbous inner segments compared to the true cones, and some ultrastructural features of their outer segments that resemble those of rods (Schott et al. 2016). These results combined demonstrate that in diurnal caenophidian snakes, rods were not lost but underwent evolutionary changes, acquiring a gross morphology similar to that of cones. Whether the rhodopsin is purely expressed in these transmuted, cone‐like rods or also co‐opted by true cones is still not clear. It also remains to be investigated in detail whether the synaptic terminals of transmuted, cone‐like rods also underwent morphological and/or physiological transformations (Underwood 1968).

In this study, we aimed to fill some important knowledge gaps regarding the outer‐retinal photoreceptor morphology and patterns of RH1 expression in “all‐cone” retinas of diurnal caenophidian snakes. Using IHC, we labeled the three visual opsins known to occur in snakes (SWS1, RH1, and LWS) in retinal sections of diurnal and nocturnal endoglyptodontan caenophidian snakes, and we assessed the distribution of photoreceptors and patterns of opsin expression in whole retinas of diurnal species. In nocturnal taxa, we identified distinct RH1 rods and (single) SWS1 and (single and double) LWS cones. Coexpression of the two cone opsins (SWS1 and LWS) in a single cone was common in some nocturnal species. Surprisingly, in diurnal caenophidians, we found that RH1 is frequently coexpressed with SWS1, something not reported elsewhere among vertebrates.

## 2 Material and Methods

### 2.1 Animals

Snakes used in this study (*n* = 32) (Table 1; Table S1) were collected through fieldwork, donated by the Butantan Institute (São Paulo, Brazil), housed at the Liverpool School of Tropical Medicine (UK), or obtained commercially. The permit for specimen collection in the field was issued by the Brazilian Ministry of the Environment and the competent authority, the Chico Mendes Institute for Biodiversity Conservation (SISBIO 79155, 86246). Animals were euthanized during the daytime, using either a lethal injection of sodium thiopental (100 mg/kg), a lethal overdose of sodium pentobarbital, or a lethal dose of ketamine and xylazine. All procedures were in accordance with ethical principles of animal management and experimentation established by the Brazilian Council for Control of Animal Experimentation (CONCEA) and approved by the Ethics Committee of Animal Research of the Psychology Institute, University of São Paulo, Brazil (permission number 9284040521); the Institutional Animal Care and Use Committee at Charles University in Prague and the Ministry of Culture of the Czech Republic (permission number UKPRF/28830/2021); or the UK Home Office and the LSTM Animal Welfare and Ethical Review Board (establishment license X20A6D134).

**TABLE 1:** Caenophidian snakes collected for morphological analyses of retinal structure and patterns of visual‐opsin expression. See Supporting Information for details of specimens.

Family	Species	Diel activity	Habit	Retinal Sections	Wholemount (# retinas)	EM	#Individuals
Viperidae	*Bothrops jararaca* ^a^	Nocturnal	ground‐dwelling	1	—	1	1
	*Bothrops jararacussu* ^a^	Nocturnal	ground‐dwelling	1	—	—	1
Elapidae	*Naja haje*	Diurnal	ground‐dwelling	1	—	—	1
	*Naja kaouthia*	Diurnal	ground‐dwelling	—	1	—	1
Psammophiidae	*Malpolon monspesulanus*	Diurnal	ground‐dwelling	1	1	—	1
	*Psammophis elegans*	Diurnal	ground‐dwelling	1	1	—	1
Colubridae	*Chironius flavolineatus*	Diurnal	arboreal	—	1	—	1
	*Leptophis ahaetulla*	Diurnal	arboreal	1	2	—	2
	*Oxybelis fulgidus*	Diurnal	arboreal	—	2	—	1
	*Mastigodryas boddaerti*	Diurnal	ground‐dwelling	—	2	—	1
	*Pantherophis guttatus*	Diurnal	ground‐dwelling	1	—	—	1
Natricidae	*Thamnophis sirtalis*	Diurnal	ground‐dwelling	1	—	—	1
Dipsadidae	*Chlorosoma viridissimum* ^a^	Diurnal	arboreal	1	—	1	1
	*Leptodeira annulata*	Nocturnal	arboreal	1	—	—	1
	*Leptodeira tarairiu*	Nocturnal	arboreal	1	—	—	1
	*Oxyrhopus guibei* ^a^	Nocturnal	ground‐dwelling	1	—	1	1
	*Oxyrhopus trigeminus*	Nocturnal	ground‐dwelling	1	—	—	1
	*Philodryas patagoniensis* ^a^	Diurnal	ground‐dwelling	1	—	1	1
	*Pseudoboa nigra*	Nocturnal	ground‐dwelling	1	—	—	1
	*Dipsas mikanii*	Nocturnal	ground‐dwelling	1	—	1	1
	*Dipsas neuwiedi*	Nocturnal	ground‐dwelling	1	—	1	1
	*Dryophylax chaquensis* ^a^	Nocturnal^b^	ground‐dwelling	1	4	—	5
	*Dryophylax phoenix*	Nocturnal^b^	ground‐dwelling	—	1	—	1
	*Tomodon dorsatus*	Diurnal	ground‐dwelling	1	6	1	4
total				19	21	7	32

Abbreviation: EM, electron microscopy.

^a^ Small, possibly juvenile specimens.

^b^ Species classified as nocturnal based on field observations (T.B.G. and N.F.T.V., personal observation), but with “all‐cone” retinal structure.

We follow a classification of caenophidians in which major lineages sometimes referred to as subfamilies within Colubridae (e.g., Colubrinae and Dipsadinae) are instead considered as families (e.g., Colubridae and Dipsadidae, respectively, following, e.g., Zaher et al. 2019). We sampled 24 species from six caenophidian families: 15 species with simplex, “all‐cone” retinas (potentially with transmuted, cone‐like rods) from five families (Colubridae, Dipsadidae, Natricidae, Psammophiidae, and Elapidae), and nine species with duplex retinas (with morphologically typical cones and rods) from two families (Viperidae and Dipsadidae) (Table 1). Information on specimens and voucher numbers is available in Table S1. Except for six small, probably juvenile specimens of *Philodryas patagoniensis*, *Chlorosoma viridissimum*, *Bothrops jararaca*, *B. jararacussu*, *Oxyrhopus guibei*, and *Dryophylax chaquensis* (Table S1), all other individuals sampled for this study were relatively large and probably adults (according to Andrade 2021; Bellini et al. 2014; Ferreira 2013; Loebens et al. 2020, 2017; Passos 2018; Pizzato et al. 2008; Quintela and Loebmann 2019; Sazima 1992; Scartozzoni et al. 2009; Silva et al. 2020; Siqueira et al. 2013). Vernacular names of many snakes are not well‐established but are reported here for the sampled species in Table S2.

Following euthanasia, eyes were enucleated, and when possible (depending on field or lab conditions and experience of dissector), small radial incisions were made in the dorsal region for subsequent orientation. Corneas were removed, and eyecups were fixed for IHC or electron microscopy.

### 2.2 Tissue Preparation for Electron Microscopy and Image Acquisition

For electron microscopy, eyecups of seven species (Table 1) were fixed in 2% glutaraldehyde (GA) solution (Electron Microscopy Sciences) diluted in 150 mM cacodylate buffer (CB), pH 7.4, for 3 h in the dark at room temperature, then preserved in 150 mM CB at 4°C. Small pieces, approximately 1 cm^2^, were cut from the central retina, adjacent to the optic nerve. Samples were stained in a solution containing 2% osmium tetroxide, 3% potassium ferrocyanide, and 2 mM CaCl_2_ in 150 mM CB for 2 h at 4°C, followed by 1% thiocarbohydrazide (1 h at 50°C) and 2% osmium tetroxide (1 h at room temperature), and then stained with 1% aqueous uranyl acetate for 6 h at 45°C and lead aspartate for 6 h at 45°C, as described by Briggman et al. (2011). Tissues were dehydrated at 4°C through an ethanol series (70%, 90%, and 100%), transferred to propylene oxide, infiltrated at room temperature with 50%/50% propylene oxide/Epon, and then 100% Epon. Samples were embedded in medium‐hard Epon (Embed812, 26 mL; dodecenyl succinic anhydride, 15 mL; *N*‐methylaniline, 11 mL; BDMA, 1.2 mL; Electron Microscopy Sciences) and cured in embedding molds at 70°C for 48 h.

Serial sections at 40 nm were collected on wafer pieces and mounted on an aluminum pin with electrically conductive glue. High‐resolution EM micrographs were taken using a Zeiss Supra 55 scanning electron microscope operating at 5 kV with the in‐lens detector.

### 2.3 IHC (Retinal Sections and Wholemounts)

Eyes collected for IHC procedures were fixed in 4% paraformaldehyde (PFA) diluted in phosphate‐buffered saline (PBS, 10 mM, pH 7.4) for 3 h at room temperature and subsequently preserved in PBS containing 0.05% sodium azide at 4°C. Eyes from three species (*Psammophis elegans*, *Pantherophis guttatus*, and *Thamnophis sirtalis*) became available when animals were euthanized for another study (Kverková et al. 2022). These eyes were dissected, fixed in PFA 4% for 1 h, rinsed in PBS, incubated in 30% sucrose solution for 24 h, transferred to an antifreeze solution (30% glycerol, 30% ethylene glycol, 40% PBS), and stored at −20°C for further processing.

Retinas from 19 species (Table 1) were used to obtain vertical sections with a vibratome (Leica VT 1200 S). Retinas were embedded in 4% agarose, and sections at 70 µm thickness were collected and stored in PBS at 4°C. All sections from each retina were pooled, and the retinal regions of origin of each section were not recorded. For wholemount preparations, retinas were carefully dissected from eyecups. When the pigment epithelium was too firmly attached to the retina and its removal would compromise the integrity of the photoreceptors, the pigment was bleached by incubating the retina in a 10% hydrogen peroxide solution diluted in PBS for 30–60 min at 55°C prior to IHC.

Vibratome sections and whole retinas were processed free‐floating. Tissues were washed three times for 15 min in PBS, blocked with 10% normal donkey serum with 1% TritonX‐100 in PBS for 1 h, and incubated in primary antibodies diluted in PBS with 1% Triton X‐100 (Table 2). Sections were incubated overnight at room temperature, and whole retinas were incubated for three to 5 days at 4°C. In retinal sections, mixtures of three primary antibodies were used to label the three visual opsins found in snakes (SWS1, RH1, and LWS) (Table 2). In whole retinas, mixtures of anti‐SWS1 and anti‐RH1 antibodies were used to analyze the expression of these two photopigments. After incubation with primary antibodies, retinas were washed four times for 15 min each in PBS and incubated with secondary antibodies diluted in PBS with 1% Triton X‐100 for 2 h at room temperature, protected from light. Donkey secondary antibodies were conjugated with Alexa 488, Cy3, and Alexa 647 (Dianova), diluted 1:500. Retinal sections were counterstained with 4,6‐diamidino‐2‐phenylindole (DAPI) (1:10,000; Sigma‐Aldrich) to visualize nuclear layers. Sections and whole retinas were carefully mounted onto glass slides with Aqua‐Poly/Mount (Polysciences) or Vectashield (Vector Laboratories Inc., California, USA) and a coverslip. Visualizations of SWS1, RH1, and LWS expression were carried out using the same secondary antibodies as above. Secondary antibody specificity was tested by omission of the primary antibodies in retinal sections. No unspecific staining was detected.

**TABLE 2:** Primary antibodies used in this study and their sources.

Antibody	Antigen	Host, type	Dilution	Source, Cat#, RRID
Blue opsin (OPN1SW)	Raised against the last 42 amino acids of the C‐terminal of human blue opsin—NKQFQACIMKMVCGKAMTDESDTCSSQKTEVSTVSSTQVGPN	Rabbit polyclonal	1:1000	Millipore; Cat# AB5407; RRID: AB_177457
Blue opsin (OPN1SW)	Raised against a synthetic peptide with 20 amino acids of human blue opsin (SWS1)—EFYLFKNISSVGPWDGPQYH	Goat polyclonal	1:1000	Santa Cruz Biotechnology; Cat# sc‐14363; RRID: AB_2158332
Red/green opsin (OPN1LW)	Raised against the last 38 amino acids of the C‐terminal of human red/green opsins—RQFRNCILQLFGKKVDDGSELSSASKTEVSSVSSVSPA	Rabbit polyclonal	1:1000	Millipore; Cat# AB5405; RRID: AB_177456
RET‐P1 (RH1)	Raised against amino acids 4–10 (TEGPNFY) at the N‐terminus of rat rhodopsin	Mouse monoclonal	1:1000	Millipore; Cat# MAB5316; RRID: AB_2156055

Images were acquired with a confocal laser scanning microscope (Leica TCS SP8), using the 405, 488, 554, and 647 nm lines and the PMT (photomultiplier settings). Settings were chosen to avoid cross‐talk between the different lines. Micrographs were obtained using an HC PL APO 40×/1.3 or HC PL APO 63×/1.4 oil immersion objective. Data were analyzed, and images were adjusted for brightness and contrast with Fiji (Schindelin et al. 2012; RRID:SCR_002285).

### 2.4 Antibody Characterization

For visual‐opsin labeling, the following antibodies were used: (i) anti‐SWS1 opsin (goat polyclonal, sc‐14363; RRID: AB_2158332; Santa Cruz Biotechnology Inc., Heidelberg, Germany; dilution 1:1000): antibody raised in goats against a synthetic peptide with 20 amino acids of human blue opsin (SWS1); (ii) anti‐SWS1 opsin (rabbit polyclonal, AB5407; RRID: AB_177457; Millipore; dilution 1:1000): antibody raised in rabbits against the last 42 amino acids of the C‐terminal of human blue opsin; (iii) anti‐LWS opsin (rabbit polyclonal, AB5405; RRID: AB_177456; Millipore; dilution 1:1000): antibody raised in rabbits against the last 38 amino acids of the C‐terminal of human red/green opsins (LWS); and (iv) anti‐rhodopsin (RH1) (clone RET‐P1, mouse monoclonal, MAB5316; RRID: AB_2156055; Millipore, Bedford, MA; dilution 1:1000): antibody raised in mice against amino acids 4–10 (TEGPNFY) at the N‐terminus of rat rhodopsin. The specificity of the anti‐SWS1 and anti‐LWS antibodies was described previously for snakes (Bittencourt et al. 2019; Hauzman et al. 2014, 2017, 2021; Gower et al. 2019; Tashiro et al. 2022). Both anti‐SWS1 antibodies label small single‐cone photoreceptors in snakes (Tashiro et al. 2022).

### 2.5 Stereological Assessment of the Density and Distribution of Photoreceptors

The density and distribution of photoreceptors were estimated for 10 species from four families (Table 1). The number of retinas sampled for each species varied from one to six, according to specimen availability (Table 1; Table S1). To assess cell density, we applied a stereological approach based on the optical fractionator method (West et al. 1991), modified for retinal wholemounts (Coimbra et al. 2009, 2013). We used a motorized fluorescent microscope (DM5500B, Leica Microsystems, Germany), with a set of filters for Alexa Fluor 488 (excitation blue, emission green) and CY3 (excitation green, emission red), connected to a computer running the Stereo Investigator software (MicroBrightField, Colchester, VT; RRID: SCR_024705).

In the program, the coordinates of the retina contour were obtained with a 5×/NA 0.15 objective. Approximately 200 counting frames evenly spaced at a determined distance were positioned randomly, covering the entire area of the retina. The sampling grid varied according to the size of the retina. Cells were counted using a 40×/0.8 objective when lying entirely within the counting frame (100 × 100 µm) or when intersecting the acceptance lines (up and right), without touching the rejection lines (down and left) of the frame (Gundersen 1977). Specific photoreceptor types were counted first by visualizing the labeled outer segments of SWS1 and RH1 photoreceptors, labeled with a combination of anti‐SWS1 and anti‐RH1 antibodies, under fluorescent light. Photoreceptors coexpressing both of the labeled photopigments, SWS1 and RH1 (here named as multiopsin cones), were assessed by interchanging the blue/green filters for each counting frame. Thereafter, all photoreceptors were counted for each counting frame by adjusting the focus of the microscope into the inner segments, viewed under bright light. LWS cones were not labeled in wholemounted retinas; counts for them were obtained by subtracting numbers of SWS1 and RH1 (and SWS1 + RH1) photoreceptors from total photoreceptor counts. In snakes, the accessory member of double cones is slender and closely attached to the principal cone and often not visible in flatmounted retinas, so double cones and large single cones were not distinguished, and combined LWS photoreceptor counts are instead reported.

To estimate the total population of photoreceptor neurons (*N*_total_), we used the following algorithm: *N*_total_ = Σ*Q* × 1/asf, where Σ*Q* is the sum of the total number of neurons counted, and asf, the area of sampling fraction, being the ratio between the counting frame and the sampling grid (Coimbra et al. 2009). The stereological parameters used to estimate the number of photoreceptors of each retina are described in Table S3. For each cell type counted, we calculated the Scheaffer coefficient of error (CE) and considered an acceptable value of < 0.10 (Glaser and Wilson 2008).

## 3 Results

### 3.1 Retina Structure and Photoreceptor Population in Diurnal and Nocturnal Caenophidian Snakes: Types of Photoreceptors, Their Synaptic Terminals, and Patterns of Visual‐Opsin Expression

#### 3.1.1 Overall Structure of the Retinas of Diurnal and Nocturnal Caenophidian Snakes

**FIGURE 1:**
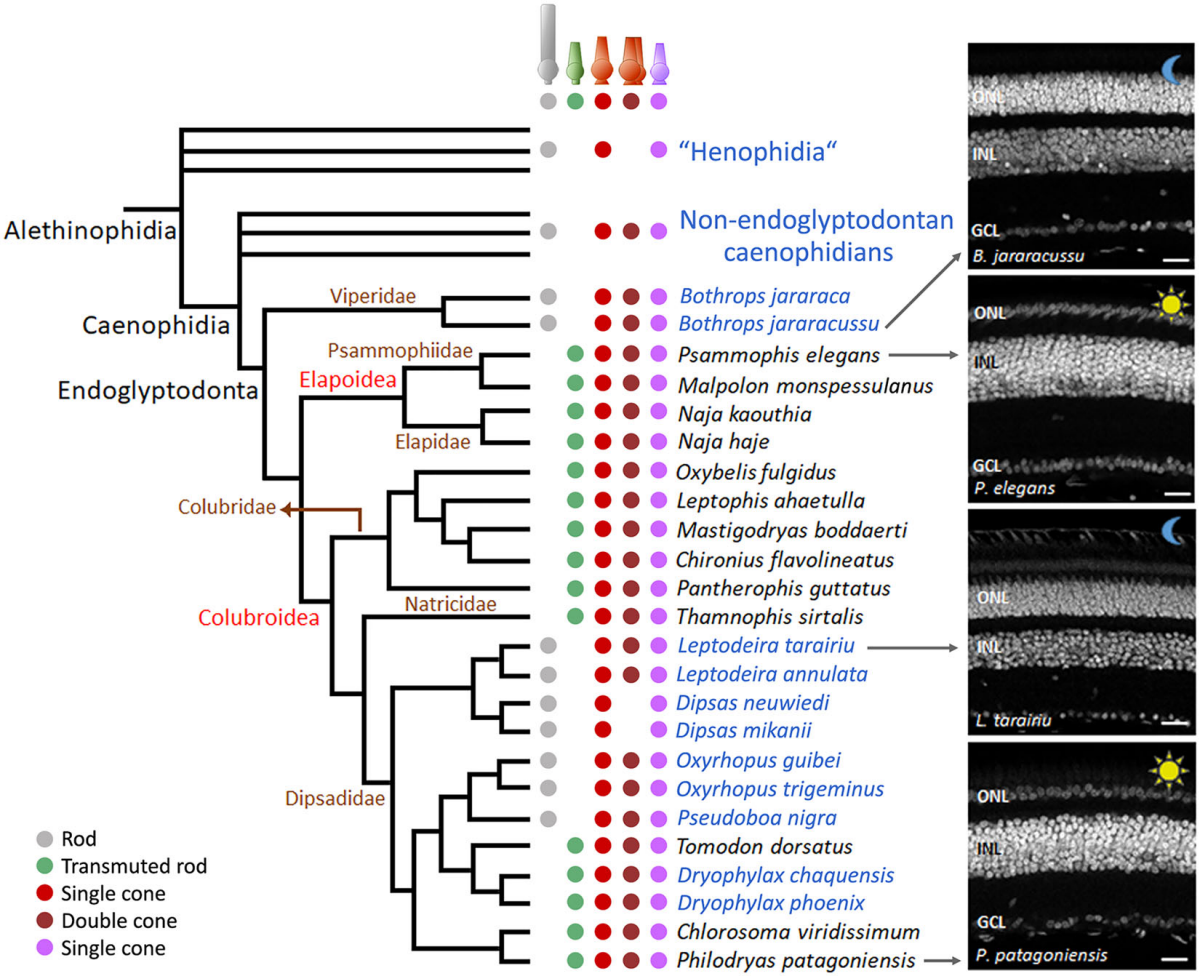
Phylogenetic relationships of the endoglyptodontan caenophidian snakes analyzed in this study, their photoreceptor complements, and the retinal structure of two diurnal and two nocturnal species are shown in DAPI‐stained cross sections. Nocturnal species are indicated in blue in the phylogenetic tree. In nocturnal species, a thick outer nuclear layer (ONL) is observed, whereas in diurnal species, the ONL has only one or two rows of photoreceptor nuclei. INL, inner nuclear layer; GCL, ganglion cell layer. Scale bars: 20 µm. Phylogenetic relationships from Pyron et al. (2013) and Zaher et al. (2009). The three parallel terminal branches depicted for “Henophidia” and for non‐endoglyptodontan caenophidians each represent an unspecified number of paraphyletic lineages. Additional information on vernacular names, types of photoreceptors, and the opsins that they (co‐)express is presented in Table S2.

**FIGURE 2:**
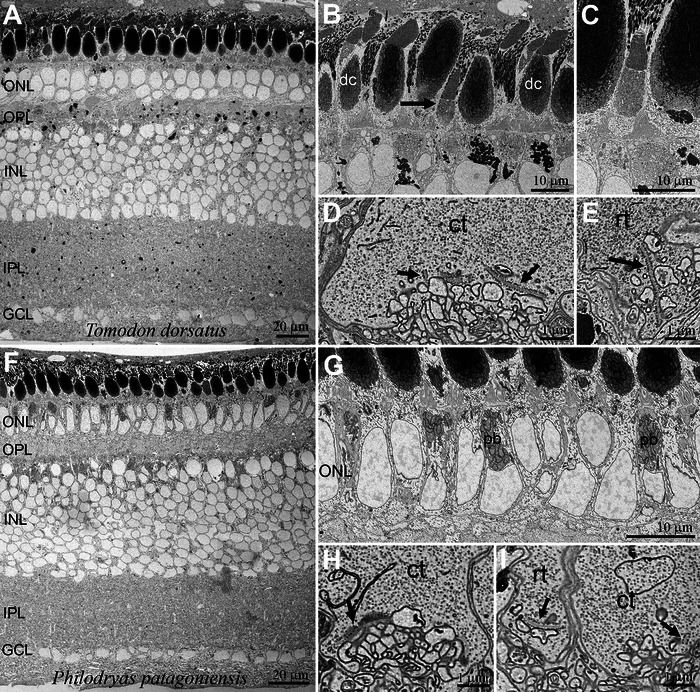
High‐resolution scanning electron microscopy (SEM) images of retinas of two diurnal dipsadid snakes: *Tomodon dorsatus* (A–E) and *Philodryas patagoniensis* (F–I). (A, F) Cross‐sections showing overviews of the retinas. (B, C, G) Details of the photoreceptor and outer nuclear layer (ONL). B. Predominance of large cones and double cones (dc), along with a distinguishable smaller photoreceptor, presumably a transmuted, cone‐like rod (arrow). The inner segments of large single cones and of the principal members of double cones appear dark due to the high concentration of electron‐dense granules in their ellipsoids. C. Higher magnification of another smaller photoreceptor, presumably a transmuted, cone‐like rod. G. Detail of the ONL, showing photoreceptor nuclei and paranuclear bodies (pb), an aggregate of mitochondria belonging to the accessory member of the double cones. (D, E, H, I) Synaptic terminals of photoreceptors, with synaptic ribbons (arrows). Typical large terminals of cones (ct) (D, H, I) and smaller, spherule‐like terminals, presumably of transmuted, cone‐like rods (rt), with a single long ribbon (E, I). GCL, ganglion cell layer; INL, inner nuclear layer; IPL, inner plexiform layer; OPL, outer plexiform layer.

**FIGURE 3:**
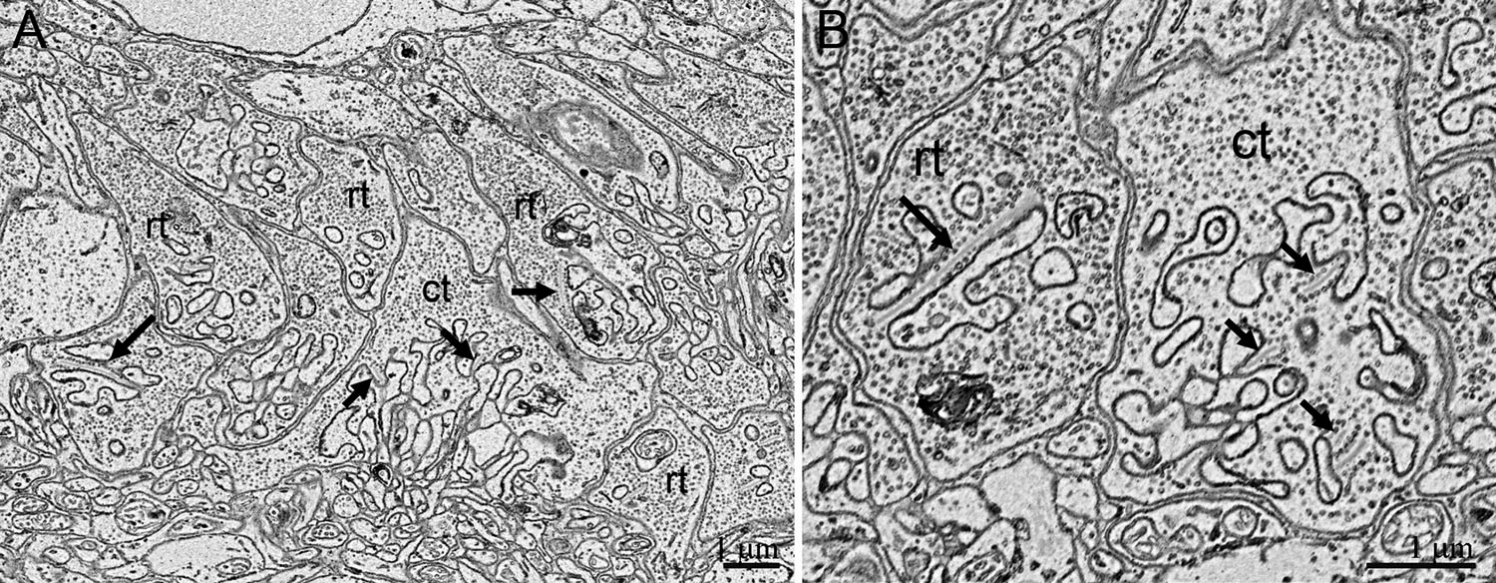
High‐resolution scanning electron microscopy (SEM) images of cross‐sectioned retinas showing the outer plexiform layer of the nocturnal dipsadid caenophidian snake *Oxyrhopus guibei*. In (A), one large cone terminal (ct) is surrounded by many rod terminals (rt). Synaptic ribbons are indicated by arrows. (B) A cone terminal (ct) with three synaptic ribbons (arrows) and a rod terminal (rt) with a single long synaptic ribbon (arrow).

**FIGURE 4:**
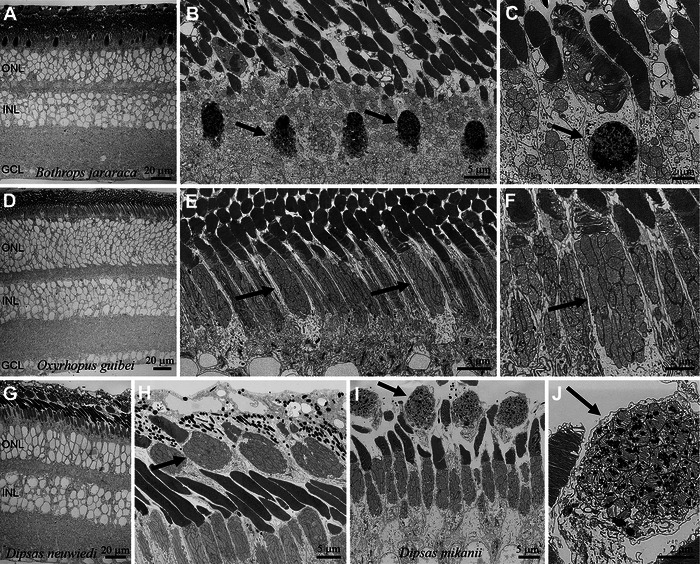
High‐resolution scanning electron microscopy (SEM) images of duplex retinas of four nocturnal caenophidian snakes. (A–C) The viperid *Bothrops jararaca* has cones with large inner segments, with ellipsoids containing mitochondria filled with electron‐dense granules (“microdroplets,” arrows). (D–F) Cones of the dipsadid *Oxyrhopus guibei* are elongated and lack microdroplets (arrows). (G–J) Species of the dipsadid genus *Dipsas* have “two‐tiered” retinas, with the inner segments of cones (arrows) lying scleral to rods; in *D. mikanii*, the ellipsoids of cones contain sparse microdroplets (arrows in I, J), which are absent in *D. neuwiedi* (arrow in H).

**FIGURE 5:**
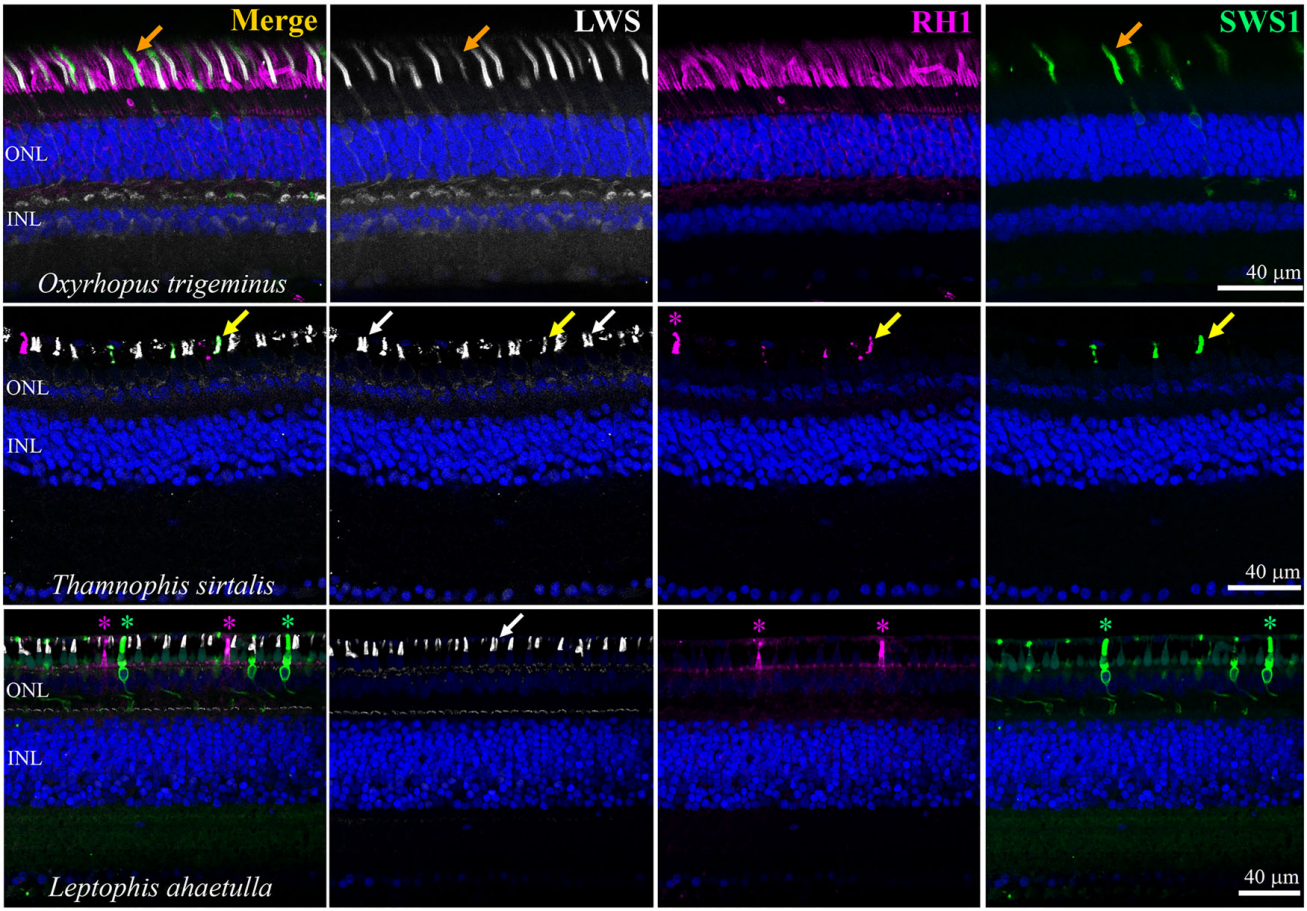
Retinal sections of nocturnal (top row) and diurnal (center and bottom rows) caenophidian snakes and patterns of visual opsin expression. In the nocturnal dipsadid *Oxyrhopus trigeminus* (top), the retina is dominated by typical rods expressing RH1 (magenta). The cone population is predominantly of LWS cones (white), with fewer SWS1 cones (green). In some nocturnal caenophidians, coexpression of the cone opsins SWS1 and LWS was observed in presumably primarily SWS1 cones (orange arrows). In the diurnal caenophidian snakes examined (center, bottom), there are no morphologically typical rods, and the RH1 (magenta) is expressed instead in a group of small, probably transmuted, cone‐like rods (magenta asterisks). The retinas of these diurnal species are dominated by LWS cones (white), both single and double (white arrows). In some species, SWS1 cones (green) frequently also coexpress RH1, and in some cases, as in the natricid *Thamnophis sirtalis* (center), all three opsins are coexpressed in some (presumably primarily SWS1) multiopsin cones (yellow arrows). In the retinal section of the colubrid *Leptophis ahaetulla* (bottom), only pure‐SWS1 cones (green asterisks) and pure‐RH1 cone‐like rods (magenta asterisks) were observed, without visual‐opsin coexpression. Neuron nuclei were stained in blue by DAPI. ONL, outer nuclear layer; INL, inner nuclear layer.

Several notable differences were observed between the retinas of sampled diurnal and nocturnal caenophidian snakes (Figures 1, 2, 3, 4, 5; Figure S1). First, an extreme difference in photoreceptor density is evidenced by the thickness of the outer nuclear layer (ONL), which comprises the nuclei of rods and cones. Confocal and electron microscopy images (Figures 1, 4, and 5; Figure S1) show approximately 5–8 rows of photoreceptor nuclei in nocturnal species. The inner nuclear layer (INL) (with the nuclei of horizontal cells, bipolar cells, and amacrine cells) is similar in thickness to or thinner than the ONL (Figures 1, 4, and 5; Figure S1), reflecting a probable high convergence of photoreceptors to second‐order neurons (bipolar cells). On the other hand, in diurnal species, a thin ONL is formed by only one or two rows of photoreceptor nuclei, and a thicker INL (Figures 1, 2, and 5; Figure S1) reflects a probable low convergence of photoreceptors to bipolar cells.

#### 3.1.2 Photoreceptor Inner Segments and Synaptic Terminals: Rod‐Like Spherules in the “All‐Cone” Retinas of Diurnal Caenophidian Snakes

From scanning electron microscopy (SEM) examinations of three diurnal dipsadids, *Tomodon dorsatus*, *Chlorosoma viridissimus*, and *Philodryas patagoniensis*, the inner segments of large single cones and the principal member of double cones have ellipsoids with large mitochondria containing a substantial amount of electron‐dense granules in their cristae (Figure 2). These granules are absent in the accessory members of double cones and in the small single cones of *T. dorsatus* and *P. patagoniensis* (Figure 2). In *C. viridissimus*, one population of small cones has sparse granules within the mitochondria of the ellipsoid (images not shown). In *T. dorsatus*, two populations of small single cones were clearly distinguishable from each other: one type is very similar in shape to large single cones, but smaller and the second type has narrower inner and outer segments (Figure 2B,C). In *P. patagoniensis*, the accessory members of double cones have pronounced paranuclear bodies, a unique structure of the ophidian double cone, composed of a second aggregation of mitochondria close to the nucleus (Underwood 1968; Walls 1942) (Figure 2F,G). The outer plexiform layer (OPL) is dominated by large pedicles of cones with numerous, long synaptic ribbons, and, in rare cases, we identified a smaller, spherule‐like terminal with a single, long synaptic ribbon and with more densely packed synaptic vesicles than in the large cone pedicles (Figure 2D,E,H,I).

In the sampled nocturnal caenophidians, with duplex retinas, rods outnumber cones, and their terminals are clearly distinguishable (Figures 3 and 4). One large cone pedicle with several small ribbons is usually surrounded by several small rod spherules with single long ribbons (Figure 3). Rods usually display a stereotypical morphology, with long and narrow inner and outer segments (Figure 4). In contrast, SEM examination of four species revealed considerable variation in cone morphology. In the viperid *Bothrops jararaca*, single and double cones have bulbous inner segments with large mitochondria in their ellipsoids containing many highly electron‐dense granules (Figure 4A–C). The outer segments of the cones are short and conical, and the outer and inner segments are positioned at the level of the outer and inner segments of the rods. On the other hand, the cones of the dipsadid *Oxyrhopus guibei* have long and narrow inner and outer segments, approaching the shape of those of the rods (Figure 4D–F). The cone ellipsoids are not bulky and are discretely larger than those of rods, and no granules were observed in their mitochondria (arrows in Figure 4E,F). In two dipsadid species of the genus *Dipsas*, the cone myoids are extremely long, in a way that their bulbous inner segments lie beyond (scleral to) the rod outer segments (Figure 4G–J). In *Dipsas mikanii*, the ellipsoids have some sparse granules within the mitochondria (Figure 4I,J), while in *D. neuwiedi*, no granules were observed (Figure 4G,H).

#### 3.1.3 Patterns of Visual‐Opsin Expression in Retinas of Caenophidian Snakes

In the duplex retinas of nocturnal viperid and dipsadid species, RH1 is expressed only in morphologically typical rods with long outer segments (Figure 5). In these taxa, the anti‐LWS antibody labeled the outer segments of most cones, and the anti‐SWS1 labeled a less abundant population of single cones (Figure 5). In nocturnal dipsadid species, coexpression of SWS1 and LWS was frequently observed in what are presumably primarily SWS1 cones, based on strong SWS1 and weak LWS labeling (Figure 5). In sampled diurnal caenophidian species, the photoreceptor population is dominated by LWS cones, both single and double (Figure 5). Two populations of small cone‐like photoreceptors were labeled with antibodies for either SWS1 or RH1 (Figure 5), the latter possibly being transmuted cone‐like rods. Coexpression of the SWS1 and RH1 opsins in a third population of small, single, cone‐like multiopsin photoreceptors was common in most species. Occasionally, in some diurnal caenophidians, we also observed some coexpression of LWS and RH1, of LWS and SWS1, and in three species from different families (*Psammophis elegans*, *Thamnophis sirtalis*, and *Tomodon dorsatus*), all three visual opsins were found to be coexpressed in a single (cone‐like) multiopsin photoreceptor, with weak labeling of LWS and RH1 and stronger SWS1 (Figure 5). See Table S2 for detailed patterns of opsin expression observed in each species analyzed.

### 3.2 Retinal Specializations: Visual‐Opsin Expression in “All‐Cone” Retinas of Caenophidian Snakes

#### 3.2.1 Patterns of RH1 and SWS1 Expression in “All‐Cone” Retinas

**FIGURE 6:**
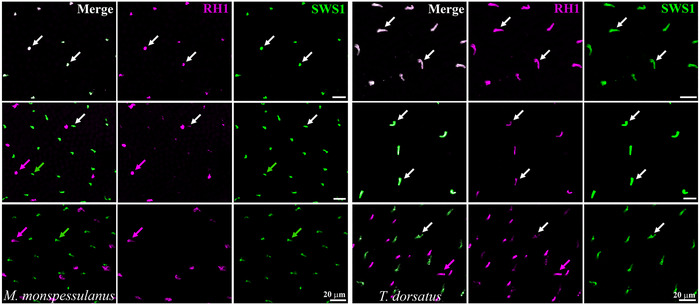
Wholemounted retina of the diurnal psammophiid *Malpolon monspessulanus* (left) and the diurnal dipsadid *Tomodon dorsatus* (right), immunolabeled with anti‐RH1 (magenta) and anti‐SWS1 (green). The images were obtained from three different regions of the same retinas and show the outer segments of the photoreceptors, with varying degrees of coexpression of the two photopigments. In the upper row, there is strong labeling of both opsins (white arrows) in multiopsin cones (coexpression appears paler in the overlaid images). In the center row, multiopsin cones express the SWS1 opsin more intensely, with weaker RH1 labeling (white arrows); in *M. monspessulanus*, some photoreceptors expressing only SWS1 (green arrow) or only RH1 (magenta arrow) are also present. Bottom row, in *M. monspessulanus*, absence of multiopsin cones (no opsin coexpression), and presence only of pure‐SWS1 cones (green arrow) and pure‐RH1 cone‐like rods (magenta arrow), and in *T. dorsatus*, all SWS1 cones show some expression of RH1 (absence of pure‐SWS1 cones), and photoreceptors expressing RH1 vary from intense labeling in pure‐RH1 cone‐like rods (magenta arrow) to weak RH1 labeling in multiopsin, primarily SWS1 cones (white arrow).

From examining immunolabeled retinas, we found that coexpression of rhodopsin with the SWS1 cone opsin is common in some of the cone photoreceptors of most diurnal caenophidian species sampled (Figure 5; Table S2). In wholemounted retinas, we observed that the relative degree of expression of the two photopigments varied among species (Figure 6). Besides pure‐SWS1 and pure‐RH1 photoreceptors, multiopsin cones (with both SWS1 and RH1) had variable degrees of RH1 expression, revealed by graded labeling intensities, from faint to strong, depending on the retinal region. In contrast, SWS1 expression appeared constant across the whole retina, as shown by intense labeling in all marked cells (Figure 6). This suggests that, along with pure‐RH1 (transmuted, cone‐like rod) photoreceptors, rhodopsin is also co‐opted by some primarily SWS1 cones in a graded manner.

#### 3.2.2 Density and Distribution of Photoreceptors: SWS1, RH1, and SWS1 + RH1 Multiopsin Cones

Using a stereological approach, we analyzed the density and distribution of photoreceptors in wholemounted retinas and the expression patterns of SWS1 and RH1 of ten endoglyptodontan caenophidian species that have “all‐cone” retinas with transmuted, morphologically cone‐like rods, including four colubrids, three dipsadids, two psammophiids, and one elapid (Tables 1 and 3). We compared the retinal topography of primarily arboreal versus primarily ground‐dwelling species, given that habit might be associated with different types of retinal specializations (Tashiro et al. 2022) (Table 1). Eight of the ten species analyzed are uncontroversially primarily diurnal. Two dipsadid species, *Dryophylax chaquensis* and *D. phoenix*, are considered primarily nocturnal and are typically found active at night (N.F.T.V. and T.B.G., personal observations) (Carrillo 2017; Guedes et al. 2014; Marques et al. 2017). However, the structure of their retinas and the nature of their photoreceptor populations are much more similar to those of diurnal species (Figure S2), and their retinas were analyzed as such. The number of retinas analyzed per species varied from 1 to 6, depending on specimen availability (Tables 1 and 3).

**TABLE 3:** Stereological assessment of total photoreceptors, SWS1 cones, RH1 photoreceptors (rods or transmuted, cone‐like rods), SWS1 + RH1 multiopsin cones, and LWS cones (single and double cones combined) in retinas of diurnal caenophidian snakes.

			Photoreceptors			SWS1 cones				RH1				SWS1 + RH1 multiopsin cones				LWS cones		
Species	Retinas	Retinal area	Estimated population	CE	Mean density	Estimated population	CE	Mean density	%	Estimated population	CE	Mean density	%	Estimated population	CE	Mean density	%	Estimated population	Mean density	%
*Tomodon dorsatus*	#1‐RE	22.3	314,699	0.007	14,112	18,401	0.03	825	5.8	26,134	0.02	1172	8.3	14,658	0.03	657	4.7	284,822	12,772	90.5
	#1‐LE	22	295,091	0.007	13,413	18,282	0.03	831	6	20,791	0.02	945	7	9881	0.03	449	3.3	265,899	12,086	90.1
	#2‐RE	30.4	257,418	0,008	8468	10,332	0.04	340	4	10,115	0.04	252	3.9	5501	0.06	181	2.1	267,054	8843	93.2
	#2‐LE	30.4	257,418	0,008	8468	10,332	0.04	340	4	10,115	0.04	252	3.9	5501	0.06	181	2.1	242,471	8057	94.2
	#3	17.5	228,208	0.007	13,041	9910	0.03	566	4.3	15,566	0.03	890	6.8	8972	0.03	512	3.9	211,704	12,097	92.8
	#4	24.3	261,621	0.007	10,766	10,397	0.04	428	4	25,880	0.02	1065	9.9	8565	0.04	353	3.3	233,908	9626	89.4
Mean ± *SD*		24.5 ± 5	273,910 ± 30,921		11,547 ± 2308	13,419 ± 3987		571 ± 212	5 ± 1	18,610 ± 6711		793 ± 366	6.8 ± 2.2	9095 ± 3136		397 ± 179	3.3 ± 0.9	250,976 ± 26,581	10,580 ± 1.984	91.7 ± 1.9
*Dryophylax chaquensis*	#1	15.6	150,085	0.008	9621	10,927	0.03	701	7.3	5063	0.04	325	3.4	1921	0.06	123	1.3	136,016	8719	90.6
	#2	29.1	189,297	0.008	6505	8456	0.04	291	4.5	10,775	0.04	370	5.7	7206	0.04	247	3.8	177,273	6092	93.6
	#3	11.2	160,761	0.007	14,354	8044	0.03	718	5	6532	0.03	583	4.1	1653	0.07	148	1	147,838	13,200	92
	#4	22.2	249,038	0.008	11,218	15,320	0.03	690	6.2	17,584	0.03	792	7.1	13,100	0.03	590	5.3	229,234	10,326	92
Mean ± *SD*		19.5 ± 7.8	187,295 ± 44,365		10,424 ± 3270	10,687 ± 3341		600 ± 207	5.7 ± 1.3	9988 ± 5613		517 ± 215	5.0 ± 1.7	5970 ± 5397		277 ± 216	2.8 ± 2	172,590 ± 41,556	9584 ± 2976	92.1 ± 1.2
*Dryophylax phoenix*	#1	23.3	208,993	0.007	8970	7798	0.04	335	3.7	6531	0.04	280	3.1	3542	0.05	152	1.7	198,205	8507	94.8
*Naja kaouthia*	#1	65	571,119	0.009	8787	39,962	0.03	615	7.0	44,505	0.03	685	7.8	299	0.3	5	0.1	486,951	7492	85.3
*Psammophis elegans*	#1	40.6	712,286	0.008	17,544	34,480	0.02	849	4.8	22,623	0.02	557	3.2	1636	0.1	40	0.2	656,819	16,178	92.2
*Malpolon monspessulanus*	#1	87.8	1,112,700	0.007	12,673	40,650	0.04	463	3.7	18,821	0.05	214	1.7	9702	0.07	111	0.9	1,062,931	12,106	95.5
*Mastigodryas boddaerti*	#1‐RE	50.6	508,056	0.009	10,041	25,902	0.03	512	5.1	31,965	0.03	632	6.3	14,244	0.04	282	2.8	464,433	9179	91.4
	#1‐LE	48.5	523,384	0.007	10,791	17,082	0.04	352	3.3	19,897	0.03	410	3.8	15,662	0.04	323	3.0	502,067	10,352	95.9
Mean ± *SD*		49.6 ± 1.5	515,720 ± 10,839		10,416 ± 531	21,492 ± 6237		432 ± 113	4.2 ± 1.3	25,931 ± 8533		521 ± 157	5 ± 1.8	14,953 ± 1003		302 ± 29	2.9 ± 0.1	483,250 ± 26,612	9765 ± 830	93.7 ± 3.2
*Chironius flavolineatus*	#1	29.9	471,678	0.006	15,775	28,963	0.03	969	6.1	14,932	0.04	499	3.2	5278	0.06	177	1.1	433,060	14,484	91.8
*Leptophis ahaetulla*	#1	23	532,356	0.004	23,146	48,418	0.01	2105	9.1	12,526	0.03	545	2.4	2270	0.07	99	0.4	473,682	20,595	89.0
	#2	23.6	507,980	0.005	21,525	47,585	0.02	2016	9.4	7597	0.04	322	1.5	53	0.5	2,2	0	452,851	19,189	89.1
Mean ± *SD*		23.3 ± 0.4	520,168 ± 17237		22,335 ± 1147	48,001 ± 589		2061 ± 63	9.2 ± 0.2	10,061 ± 3485		433 ± 158	1.9 ± 0.6	1161 ± 1.567		51 ± 68	0.2 ± 0.3	463,267 ± 14,730	19,892 ± 995	89.1 ± 0.1
*Oxybelis fulgidus*	#1‐RE	36.1	1,036,740	0.004	28,719	65,987	0.02	1828	6.4	16,304	0.03	452	1.6	2149	0.09	60	0.2	956,598	26,499	92.3
	#1‐LE	36	997,001	0.004	27,695	63,240	0.02	1757	6.3	13,200	0.04	367	1.3	34	0.7	1	0	920,595	25,572	92.3
Mean ± *SD*		36.1 ± 0.1	1,016,871 ± 28,100		28,207 ± 724	64,614 ± 1943		1792 ± 50	6.4 ± 0	14,752 ± 2,195		409 ± 60	1.4 ± 0.2	1092 ± 1495		30 ± 41	0.1 ± 0.1	938,596 ± 25,457	26,035 ± 655	92.3 ± 0

*Note:* LWS was not labeled in these wholemount preparations, so the LWS cone populations were estimated by subtracting SWS1, RH1, and SW1 + RH1 photoreceptors from total photoreceptor counts. The lack of LWS labeling also means that some of the SWS1 + RH1 multiopsin cones might also express LWS in at least some taxa. Mean densities in cells in mm^−2^.

Abbreviations: CE, Scheaffer's coefficient of error; LE, left eye; RE, right eye; *SD*, standard deviation.

**FIGURE 7:**
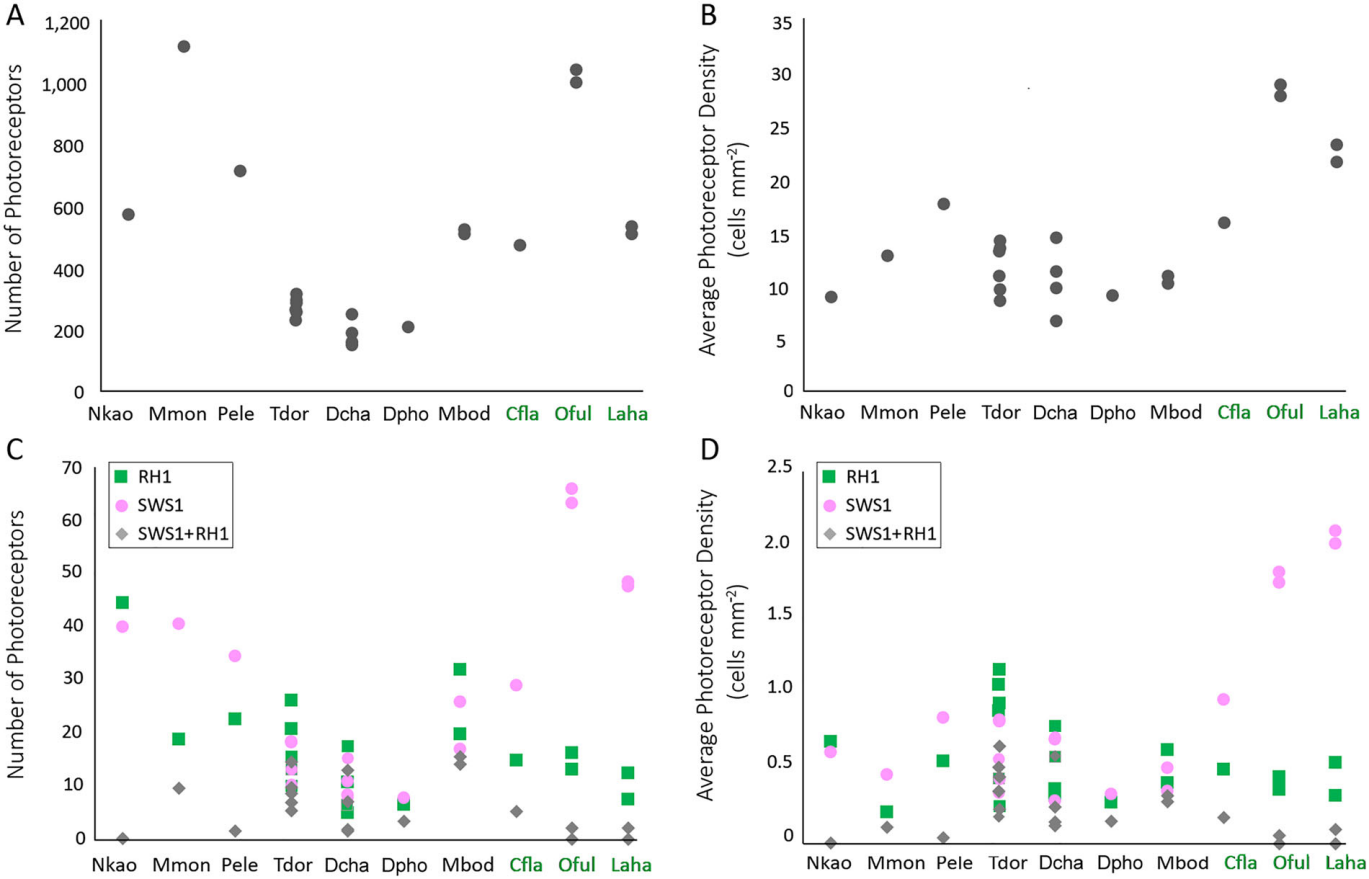
Total number and mean density of all photoreceptors (A, B) and photoreceptor subtypes (C, D) in retinas of caenophidian snakes: RH1 photoreceptors (green squares), SWS1 cones (magenta circles), and SWS1 + RH1 multiopsin cones (grey diamonds). The values should be multiplied by 10^3^. Cfla, *Chironius flavolineatus*, Dcha, *Dryophylax chaquensis*, Dpho, *Dryophylax phoenix*, Laha, *Lepthophis ahaetulla*. Mbod, *Mastigodryas boddaerti*, Mmon, *Malpolon monspessulanus*, Nkao, *Naja kaouthia*, Oful, *Oxybelis fulgidus*, Pele, *Psammophis elegans*, Tdor, *Tomodon dorsatus*, Arboreal species are indicated in green text; other species are ground‐dwelling.

**FIGURE 8:**
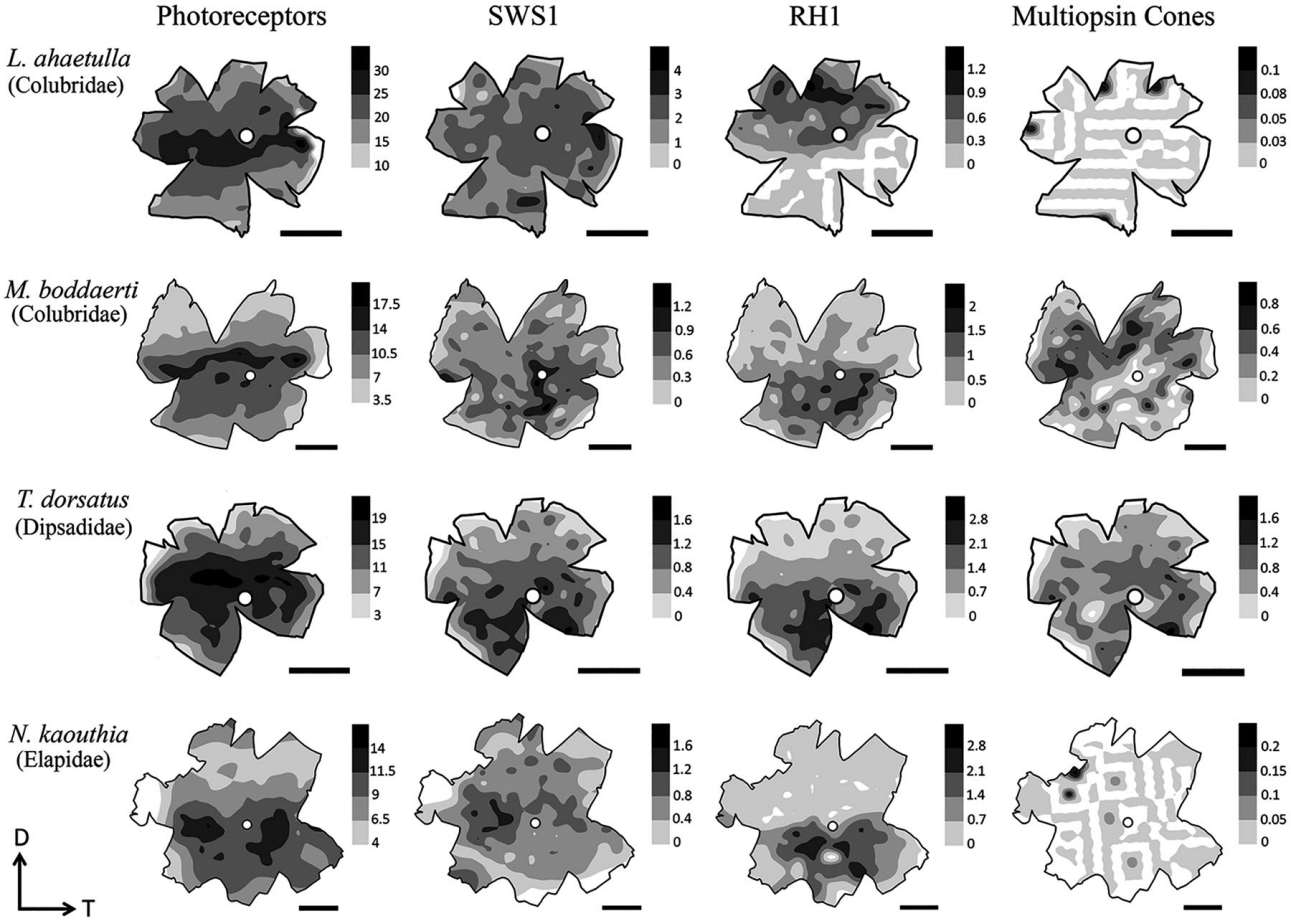
Representative topographic maps of the retinas of four caenophidian snakes: the arboreal colubrid *Leptophis ahaetulla* and the ground‐dwelling colubrid *Mastigodryas boddaerti*, dipsadid *Tomodon dorsatus*, and elapid *Naja kaouthia*, showing different distribution patterns of total photoreceptors, SWS1‐expressing cones (pure‐SWS1 and SWS1 + RH1 multiopsin cones), RH1‐expressing photoreceptors (pure‐RH1 cone‐like rods and SWS1 + RH1 multiopsin cones), and SWS1 + RH1 multiopsin cones. Topographic maps of the other six sampled species are found in Figure S3. Gray shaded bars indicate the number of cells mm^−2^. The values should be multiplied by 10^3^. The optic nerve head is depicted as a white circle. D, dorsal; T, temporal.

Mean density of total photoreceptors varied from ca. 9000–12,000 cells mm^−2^ in most ground‐dwelling species to ca. 28,000 cells mm^−2^ in the highly arboreal colubrid *Oxybelis fulgidus* (Table 3, Figure 7). Isodensity maps of the total photoreceptor complements showed a visual streak in nine of the ten snake species (Figure 8; Figure S3). In some species, the visual streak was well‐defined, extending in a relatively tapered manner along the nasal‐temporal axis (e.g., the arboreal and ground‐dwelling colubrids *Leptophis ahaetulla* and *Mastigodryas boddaerti*, respectively; Figure 8; Figure S3). In other species, the visual streak was less defined, extending to the ventral quadrant of the retina (e.g., the ground‐dwelling psammophiid *Psammophis elegans*; Figure S3). In only one species, the ground‐dwelling elapid *Naja kaouthia*, was no distinct specialization observed (no visual streak or *area centralis*), and the photoreceptors were concentrated in the ventral retina, with a peak density in the temporal region (Figure 8).

The number of SWS1‐expressing cones (including pure‐SWS1 cones and SWS1 + RH1 multiopsin cones) ranged from approximately 4% to 9% of the total photoreceptor population, depending on species (Table 3, Figure 7). The distribution of these cones varied considerably among species (Figure 8; Figure S3). In the three arboreal colubrids *Oxybelis fulgidus*, *Leptophis ahaetulla*, and *Chironius flavolineatus*, a diffuse distribution throughout the retina was observed, with higher densities in the temporal retina. Among the ground‐dwelling species, in the elapid *Naja kaouthia* and the psammophiid *Malpolon monspessulanus*, higher density was also observed in the temporal retina. A poorly defined *area centralis* was located in the ventral retina in the psammophiid *Psammophis elegans*, the dipsadid *Tomodon dorsatus*, and the colubrid *Mastigodryas boddaerti*. In general, the distribution of pure‐SWS1 cones was similar to that of total SWS1 cones, but in a more delimited manner (Figure S3). In the dipsadids *Dryophylax chaquensis* and *D. phoenix*, SWS1‐expressing cones overall had a diffuse distribution, but pure‐SWS1 cones were concentrated in a central and ventral area (Figure S3).

The proportion and distribution of RH1‐expressing photoreceptors (including transmuted cone‐like rods and SWS1 + RH1 multiopsin cones) varied considerably among arboreal and ground‐dwelling species. Highly arboreal colubrids had fewer RH1 photoreceptors (e.g., ca. 1.4% of the photoreceptor population in *O. fulgidus*), while more were observed in some ground‐dwelling dipsadids and elapids (ca. 7%–8% in *Tomodon dorsatus* and *Naja kaouthia*) (Table 3, Figure 7). In the three arboreal colubrids, *O. fulgidus*, *L. ahaetulla*, and *C. flavolineatus*, RH1‐photoreceptors were concentrated in a dorsal *area centralis* (Figure 8; Figure S3). In contrast, in ground‐dwelling species (elapids, dipsadids, psammophiids, and colubrids), these photoreceptors were concentrated in a ventral *area centralis* (Figure 8; Figure S3), with only two exceptions: the psammophiid *M. monspessulanus* had a higher density in the ventral retina but without a defined *area* (rather, a C‐shaped, central–ventral arc), and the dipsadid *D. phoenix* had a diffuse distribution without any specialization. The distribution of pure‐RH1 photoreceptors showed a similar distribution compared to the total RH1 photoreceptors, but in a more defined manner. For example, in *M. monspessulanus*, pure‐RH1 photoreceptors formed a temporal *area*, and in *D. phoenix*, these cells were concentrated in the ventral retina, as in other ground‐dwelling species (Figure S3).

The proportion of multiopsin cones coexpressing SWS1 and RH1 varied considerably, from < 0.2% of the total photoreceptor population in some arboreal (*O. fulgidus*, *L. ahaetulla*) and ground‐dwelling (*N. kaouthia*, *P. elegans*) species, with distribution restricted to some isolated points in the dorsal or ventral retina, to ca. 3% of the photoreceptors in the ground‐dwelling *T. dorsatus*, *D. chaquensis*, and *M. boddaerti* (Table 3; Figures 7 and 8; Figure S3). These SWS1 + RH1 multiopsin cones showed, in general, a diffuse distribution throughout the retina, with no defined specialization. In *C. flavolineatus*, SWS1 + RH1 multiopsin cones were more concentrated in the central retina, and in *M. monspessulanus*, in the ventral region; in *M. boddaerti*, they were more dense in the dorsal retina, in contrast to the distribution of pure‐SWS1 and pure‐RH1 photoreceptors, while in *T. dorsatus* and *D. chaquensis*, SWS1 + RH1 multiopsin cones had higher densities in the ventral retina, as also for pure‐SWS1 and pure‐RH1 photoreceptors (Figure 8; Figure S3).

In the “all‐cone” retinas of the analyzed species, the photoreceptor population is dominated by single and double LWS cones. The percentage of total LWS cones ranged from approximately 85% of the photoreceptors in the retina of *Naja kaouthia* to 96% in the retina of *Mastigodryas boddaerti* (Table 3).

## 4 Discussion

### 4.1 Retinal Structure of Diurnal and Nocturnal Caenophidian Snakes

In 13 primarily diurnal snake species from five families (Table 1, Figure 1), we observed an “all‐cone” retinal pattern, characterized by the absence of morphologically typical rods (i.e., with narrow, elongated outer segments) and the presence of only cone‐like photoreceptors with short, conical outer segments. These retinas have a thin ONL with only one or two rows of photoreceptor nuclei and a thick INL, indicating probable low convergence of photoreceptors to bipolar cells, a typical feature of photopic visual systems, with low sensitivity to light but high spatial resolution (Walls 1942). In nine out of 11 of the sampled species classified as primarily nocturnal (Table 1, Figure 1), we observed a morphologically duplex retina, containing both cones and typical rods, along with a thick ONL with multiple rows of photoreceptor nuclei. In contrast, these retinas exhibit a slightly thinner INL, indicating probably high convergence of photoreceptors to bipolar cells, a hallmark of a highly sensitive, scotopic system (Walls 1942).

Two species of the genus *Dryophylax* (*D. chaquensis* and *D. phoenix*), although primarily nocturnal (T.B.G. and N.F.T.V., personal observation) (Carrillo 2017; Guedes et al. 2014; Marques et al. 2017), exhibit a typical diurnal retinal pattern, inconsistent with their apparent circadian activity. A diurnal “all‐cone” retina has also been described in other species of this genus (Hauzman 2014) and for freshwater snakes of the genus *Helicops* (Hauzman et al. 2021), which are also primarily nocturnal (De Aguiar and Di‐Bernardo 2004; Martins and Oliveira 1998). It has been suggested that this incongruence between retinal structure and diel activity pattern in these two genera may be due to evolutionary shifts in activity period from recently diurnal ancestors, possibly related to hunting strategies (Hauzman et al. 2021; Torello‐Viera and Marques 2019). Species of *Helicops* and *Dryophylax* actively search for frogs near water bodies (Moraes‐da‐Silva et al. 2019; Pergentino and Ribeiro 2017), suggesting that recent adjustments in the occupied niche may not have been accompanied (yet) by corresponding changes in retinal structure and photoreceptor morphology (Hauzman et al. 2021).

Although unusual in vertebrates, the “all‐cone” retinal pattern is widespread among diurnal squamate reptiles (Walls 1942). In mammals, for instance, rods dominate the retina in most species (Peichl 2005), even in primarily diurnal groups such as primates. A few exceptions are observed, such as tree shrews, meerkats, and diurnal squirrels, which have cone‐dominated retinas, seemingly better adapted to photopic vision (Bernau 1969; Peichl 2005). In caenophidian snakes, a cone‐dominated retina appears to be the norm in diurnal species and may correspond to the ancestral state of the clade comprising the most recent common ancestor of Elapoidea and Colubroidea and all of its descendants (see Gower et al. 2022) (see below). The presence of duplex and all‐cone retinas across different caenophidian lineages further highlights the remarkable adaptive plasticity of the snake visual system.

### 4.2 Photoreceptor Morphology

Classical morphological studies classified the retinas of diurnal snakes as “all‐cone” due to the absence of morphologically typical rods, a low density of photoreceptors (Underwood 1967; Walls 1942; Wong 1989), and lack of visual purple (Walls 1932), the latter being the photopigment rhodopsin (RH1), previously identified by the observation of a lavender coloration in freshly dissected, dark‐adapted retinas. However, more recent studies using molecular biology and electron microscopy have revealed the presence of rhodopsin in “all‐cone” retinas of diurnal snakes (Simões et al. 2016) and photoreceptors with ultrastructural features typical of rods (Schott et al. 2016). In the natricid *Thamnophis proximus*, some photoreceptors exhibit the characteristic rod outer‐segment ultrastructure, with membranous discs completely detached from the plasma membrane (Schott et al. 2016), a trait that distinguishes rods from cones, the latter having outer‐segment discs connected to the cell membrane. These findings provided support for Walls' transmutation theory (Walls 1934, 1942). Thus, in diurnal snakes, rods were not lost but instead acquired gross‐morphological and possibly functional characteristics equivalent to those of typical cones, yet to be determined in electrophysiological studies (Bhattacharyya et al. 2017; Schott et al. 2016). However, other features of the photoreceptor structure, also relevant for distinguishing between rods and cones—such as their synaptic terminals—have never been systematically compared in diurnal and nocturnal snakes and were examined in this study for the first time.

### 4.3 Photoreceptor Inner Segments

Using IHC and SEM, we identified four types of cone‐like photoreceptors in diurnal snakes: large single cones, double cones, and two types of small single “cones,” consistent with previous studies (Hart et al. 2012; Hauzman et al. 2014, 2017; Schott et al. 2016). In three diurnal dipsadids (*Tomodon dorsatus*, *Chlorosoma viridissimus*, and *Philodryas patagoniensis*), SEM images revealed that large single cones and the principal member of double cones have inner‐segment ellipsoids that contain large mitochondria with highly electron‐dense granules, while the accessory member of double cones and small cones lack these structures. One clearly distinguishable type of small single cone‐like photoreceptor has narrower inner segments, and these are presumably the transmuted, cone‐like rods in diurnal snakes.

Ball et al. (2022) provided insights into the optical role of the aggregated mitochondria within the ellipsoids of mammals, showing that they act as microlens‐like features that focus light onto the outer segments. In snakes, the granules in the cone ellipsoids were previously reported in the retinas of two diurnal caenophidians, *Thamnophis sirtalis* and *Elaphe climacophora*, and characterized as “microdroplets”—small lipid structures approximately 0.1 µm in diameter (Wong 1989). These microdroplets form a highly refractive agglomerate, referred to as a “refringent body,” observed in the ellipsoid of true cones of many caenophidians (Underwood 1967). Wong (1989) suggested that these refractive microdroplets function as a condenser, directing light toward the outer‐segment discs, thus increasing photon capture by the photopigments and reducing the Stiles–Crawford effect (Bossomaier et al. 1989; Wong 1989). This mechanism may compensate for the absence of cone oil droplets in snakes (present in lizards, e.g., Underwood 1970; Walls 1942). Comparatively, in species of tree shrew (genus *Tupaia*) that possess cone‐dominated retinas, extraordinarily large mitochondria in the cone ellipsoids have cristae with unique concentric patterns arranged in a highly ordered manner (Samorajski et al. 1966), a feature believed to enhance the directional transmission of photons to the cone outer segments (Knabe et al. 1997). These megamitochondria are absent in the inner segments of *Tupaia* rods (Foelix et al. 1987).

Among diurnal caenophidian snakes, retinal structure and photoreceptor morphology appear broadly similar across species. In contrast, nocturnal species analyzed in this study show considerable variability in photoreceptor morphology. In nocturnal species with duplex retinas, rods outnumber cones and follow a typical amniote pattern, with relatively narrow and elongated inner and outer segments. However, in four species analyzed by SEM, cone morphology varied substantially (Figure 3). In the viperid *Bothrops jararaca*, cones resemble those of diurnal caenophidians, with bulbous inner segments and short, conical outer segments interspersed among rod outer segments, along with highly electron‐dense granules in the ellipsoids, indicating the presence of microdroplets. In contrast, the cones of the dipsadid *Oxyrhopus guibei* have extremely elongated, narrow inner and outer segments, nearly identical in length to those of rods, superficially resembling the photoreceptors found in mice and rats (Carter‐Dawson and LaVail 1979). The ellipsoids in these cones are slightly wider than those of rods and lack microdroplets. We speculate that the elongated inner and outer segments of *O. guibei* cones, similar to typical rods, may reduce the strong Stiles–Crawford effect associated with the much shorter outer segments in diurnal snakes, possibly explaining the absence of microdroplets in this species. In two species of the dipsadid *Dipsas*, cone myoids are unusually elongated, with bulbous inner segments positioned above the outer segments of rods, a condition that has been referred to as a “two‐tiered” retina (e.g., Underwood 1967) and observed in other species, including of the dipsadid *Leptodeira* and the viperid *Echis coloratus* (Gower et al. 2019; Miller and Snyder 1977). Interestingly, one of the two sampled species of *Dipsas* (*D. mikanii*) has sparse microdroplets in the ellipsoids, while the other (*D. neuwiedi*) lacks these structures. Further studies of this genus might help to clarify the origin and function of microdroplets.

We hypothesize that one explanation for the differences in variability between diurnal and nocturnal caenophidians might be that nocturnal habits in the lineages we sampled were reacquired multiple times independently from a diurnal ancestral endoglyptodontan caenophidian, providing selective pressure for a shift from cone‐ to rod‐dominated retinas and the reacquisition of a duplex pattern. In different lineages of nocturnal endoglyptodontan caenophidians, nocturnal adaptation was seemingly achieved either by elongating the inner and outer segments of cones to compete with rods for incident photons (as in *O. guibei*) or by elongating the myoid region, leading to “two‐tiered” retinas that would seem to somewhat separate the photopic and scotopic systems (as in *Dipsas*) (Miller and Snyder 1977). Under this interpretation, a diurnal retinal pattern was ancestral to the clade comprising non‐viperid, non‐homalopsid endoglyptodontans (i.e., Elapoidea + Colubroidea, which includes all families sampled in this study except Viperidae), and nocturnality reemerged multiple times, shaping distinct adaptive trajectories in the visual system of nocturnal caenophidians. Tests and refinement of this hypothesis would benefit from additional taxon sampling, studies of molecular genetics and inner retinal circuitry, and formal ancestral‐state reconstruction analyses. An alternative or additional explanation for the greater variability of nocturnal caenophidian snake photoreceptors might be that these species have an underappreciated diversity of visual ecologies.

Kim et al. (2016) proposed that in mammals, most rods originate from short‐wavelength sensitive (SWS) cones during development, a process not observed in other vertebrates. In mammals, rod and cone differentiation is controlled by two transcription factors, NRL (neural retina leucine zipper) and TRβ2 (thyroid hormone receptor β2) (Ng et al. 2011; Swaroop et al. 2010). Postmitotic photoreceptor precursors are predetermined to become SWS‐cones, and the presence of NRL in these precursors triggers rod differentiation (Oh et al. 2007). Future research should examine the hitherto neglected developmental mechanisms underlying photoreceptor differentiation in snakes. Like mammals, snakes likely evolved from a lineage that passed through a nocturnal, or at least scotopic, “bottleneck” (Walls, 1942; Emerling 2017; Gerkema et al. 2013) (but see Baden, 2024 and Fornetto et al. 2024, for a recently proposed alternative view), leading to similar adaptations in the visual system to low‐light conditions, including rod‐dominated duplex retinas, two types of single cones (expressing LWS and SWS1 visual opsins), and the absence of double cones and oil droplets, as seen in extant “basal” henophidian snakes. Investigating potential adaptive convergences in the molecular mechanisms of rod differentiation, particularly in diurnal and nocturnal caenophidians, could offer further valuable insights into the evolution of vertebrate vision.

### 4.4 Photoreceptor Synaptic Terminals

Based on outer‐retinal photoreceptors, Underwood (1968) proposed two types of evolutionary transformations of cells: “Walls transformation,” where one organelle gradually changes into another type, and “Pedler transformation,” where multiple types of organelles recombine in a mosaic‐like manner. In alligators, Kalberer and Pedler (1963) found a minority of cone‐like outer segments among numerous rod‐like ones, combined with many complex cone pedicles and a few simple rod terminals. They concluded that alligator retinas must therefore contain many photoreceptors with rod‐like outer segments and cone‐like pedicels. In nocturnal caenophidian snakes, we observed small rod‐like terminals (spherules) with one or two long synaptic ribbons and larger, more complex cone‐like terminals (pedicles) with many synaptic ribbons. In diurnal caenophidians (with “all‐cone” retinas), large cone pedicles predominated, with occasional small terminals resembling typical rod spherules. This is the first time that cones and transmuted, cone‐like rod synaptic terminals have been distinguished in diurnal snake retinas, providing further evidence for Walls’ transmutation theory. In diurnal caenophidian snakes, photoreceptor transmutation (of rods to cone‐like rods) appears to involve the shortening of rod outer segments and reduction of their population, while the synaptic terminal structure and parts of their phototransduction molecular genetic complement (Schott et al. 2016) remain conserved.

### 4.5 Patterns of Visual‐Opsin Expression

Immunohistochemical analyses revealed the expression of three visual opsins—SWS1, RH1, and LWS—in all caenophidian snake species sampled in this study. In the rod‐dominated retinas of nocturnal species, RH1 expression is restricted to typical rods with long outer segments, while the cone opsins SWS1 and LWS are expressed in two distinct cone populations. In diurnal species, the photoreceptor population is primarily composed of single and double LWS cones, with smaller “cones” expressing SWS1 and RH1. In all diurnal species, RH1 is expressed in photoreceptors with a cone‐like gross morphology. In the diurnal psammophiid *Malpolon monspessulanus*, previous studies failed to detect RH1 expression (Simões et al. 2016) or a photopigment with spectral sensitivity in the rhodopsin range (Govardovskii and Chkheidze 1989). However, our IHC analysis revealed RH1 expression in cone‐like photoreceptors. We suggest that future studies reexamine RH1 expression in the retina and assess the functionality of the RH1 protein in this species.

In nocturnal caenophidians, we frequently observed coexpression of the two cone opsins within the same photoreceptor, with stronger SWS1 and weaker LWS immunolabeling. This pattern suggests that in nocturnal species, the photopigment sensitive to medium/long wavelengths (LWS) is co‐opted by some UV/short wavelength cones. In most diurnal species, we observed wide coexpression of RH1 with the cone opsin SWS1 in cone‐like photoreceptors. Occasionally, we also observed coexpression of the three visual opsins in a single photoreceptor, with strong labeling of the SWS1 opsin and weaker labeling of the LWS and RH1, suggesting that the latter two photopigments are co‐opted by some primarily SWS1 cones. In the tiger salamander (*Ambystoma tigrinum*), up to three visual opsins are expressed in a single photoreceptor: UV‐sensitive cones have a primary SWS1 opsin and two secondary components, a short‐wavelength‐sensitive opsin (SWS2) and a LWS‐opsin, both expressed at levels more than 100 times lower than the level of the primary opsin (Isayama et al. 2014; Makino and Dodd 1996), suggesting that SWS2 and LWS opsins are expressed in some primarily UV cones in that species.

Based on the labeling patterns, we suggest that our results are not technical artifacts, because: (i) opsin coexpression was not detected in all species analyzed—for example, in the highly arboreal colubrids *Oxybelis fulgidus* and *Leptophis ahaetulla* it is barely observed; and (ii) when present, opsin coexpression is not uniformly distributed across the retina but follows a graded pattern that varies by retinal region. If the double labeling were due to cross‐reactivity of the antibodies, we would expect it to be more homogeneous throughout the retina, without cones expressing only one opsin. Additionally, opsin coexpression has been well documented in various other vertebrates, including fish (Dalton et al. 2017, 2014), amphibians (Isayama et al. 2014), and mammals (Applebury et al. 2000; Glösmann et al. 2008; Lukáts et al. 2002, 2005; Peichl et al. 2004; Röhlich et al. 1994), and has been confirmed through physiological methods (Calderone and Jacobs 1995; Dalton et al. 2017, 2014; Isayama et al. 2014).

The expression of two visual pigments in one photoreceptor broadens the spectral range of its sensitivity (Applebury et al. 2000). The functional relevance of this phenomenon, therefore, may be associated with an increased capacity to detect photons under photopic conditions. In contrast, the capacity for color discrimination may be impaired, depending on the levels of coexpression of the opsins. In a species of cichlid fish, Dalton et al. (2014) described different combinations of opsins with similar spectral sensitivity (RH2Aα, RH2Aβ, RH2B, and LWS) being coexpressed in different regions of the retina, resulting in regionalized spectral adjustments to environmental light. In mammals, the coexpression of SWS1 and LWS opsins has been demonstrated by IHC in several taxa, including species of marsupials, lagomorphs, and rodents (Applebury et al. 2000; Glösmann et al. 2008; Lukáts et al. 2002, 2005; Peichl et al. 2004; Röhlich et al. 1994). In some rodents, such as the Siberian hamster (*Phodopus sungorus*) and the pouched mouse (*Saccostomus campestris*), all cones express both LWS and SWS1 cone opsins with no signs of regional gradients in the levels of expression (Lukats et al. 2002). Therefore, only a single LWS‐SWS1 cone population exists, which precludes color vision (Lukáts et al. 2002). In the rod‐dominated retina of mice, although most cones coexpress both LWS and SWS1 opsins (Applebury et al. 2000), they can still discriminate colors (Jacobs et al. 2004; Denman et al., 2018; Szatko et al., 2020; Franke et al. 2024; Höfling et al. 2024). The LWS opsin in mice is expressed in a dorso‐ventral graded level, while the levels of SWS1 opsin are relatively constant (Applebury et al. 2000). In the ventral retina, only a minority of cones exclusively express SWS1 opsin and are selectively contacted by blue‐cone bipolar cells (Haverkamp et al. 2005). This organization results in a dorsal visual field with “normal” color vision and a ventral, achromatic retina that may primarily serve specific contrast‐detection tasks (Baden et al. 2013; Breuninger et al. 2011; Neitz and Neitz 2001; Yin et al. 2006). As in mice, we consider it plausible that, in diurnal snakes, photoreceptors expressing exclusively RH1 or SWS1 may serve as feature detectors for chromatic and non‐chromatic pathways (Baden 2024), while cones coexpressing both photopigments may play a role in essential non‐chromatic functions, such as contrast and luminance detection. Post‐receptoral visual processing pathways have never been examined in snake retinas, and future studies should investigate the presence of specific blue‐cone bipolar cells that could enable color vision in this group.

Although the coexpression of cone opsins appears to be very common among vertebrates, the coexpression of cone opsins with RH1, the typical rod opsin, seems to be extremely uncommon. To our knowledge, it has been described only for two species of snakes and in the diurnal ground squirrel. The burrowing snake genus *Anilios*, a member of the lineage (typhlopoid and leptotyphlopoid scolecophidians) that is a sister group to all other extant snakes, has apparently all “rod” retinas in which the LWS opsin is co‐opted by some rods (Gower et al. 2021). In the cone‐dominated retina of the ground squirrel (*Spermophilus citellus*), the SWS1 opsin is co‐opted by some rods (Szél and Röhlich 1988). Interestingly, in both the rod‐dominated retinas of scolecophidian snakes and the cone‐dominated retinas of squirrels, the cone opsins (LWS or SWS1) seem to be coexpressed with the rhodopsin in some rods. Conversely, based on the labeling patterns observed in our analyses of caenophidian snakes, we interpret that rhodopsin is co‐opted by some SWS1 cones, in addition to being expressed purely in transmuted, cone‐like rods. In diurnal caenophidian snakes, rhodopsin has been suggested to function under photopic or mesopic conditions, with a significant shift in its peak spectral sensitivity toward shorter wavelengths (484 nm), potentially contributing to diurnal color vision and compensating for the ancestral loss of RH2 opsins in snakes (Schott et al. 2016). The coexpression of an S/UV opsin with an “M opsin” (in this case, RH1) in multiopsin cones in caenophidian snakes represents an intriguing evolutionary parallel with mammals (with S + M opsin coexpression). This adaptation may play a crucial role in optimizing light capture under photopic or mesopic conditions.

### 4.6 Retinal Topography

The spatial organization of retinal neurons is not homogeneous and varies substantially not only among species but also within the same individual, depending on the cell type (Collin, 2008; Ahnelt and Kolb, 2000; Heukamp et al., 2020; Zhou et al., 2020; de Busseroles et al., 2021). This variability enables optimal sampling of the visual field and the extraction of light information necessary for executing specific behaviors (Baden et al., 2013; Qiu et al., 2021). Differences in habitat use, hunting strategies, prey type, and predation pressure are traits that appear to be associated with structural plasticity and considerable changes in the distribution of different cell types in the retina, as has been described in various vertebrate species (Collin, 1999; Schiviz et al., 2008; Coimbra et al., 2014), including some snakes (Hart et al. 2012; Hauzman et al. 2014, 2018, 2021; Tashiro et al. 2022).

In 9 out of 10 caenophidian snake species analyzed, the photoreceptors form a horizontal streak—well‐defined in some species (e.g., *Leptophis ahaetulla*, *Mastigodryas boddaerti*) and less delimited in others (e.g., *Dryophylax phoenix*, *Psammophis elegans*). This type of specialization may facilitate a panoramic view of the environment without the constant need for eye and head movements (Collin 2008), which could enhance foraging efficiency. The only exception observed was in the elapid *Naja kaouthia*, which exhibited a higher density of photoreceptors in the ventral retina. This specialization may enhance *N. kaouthia*’s superior visual field, potentially relating to its characteristic defensive behavior of raising the anterior part of its body perpendicular to the ground while slightly reclining its head backwards and displaying its “hood.” It can also “spit” venom anteriorly from its fangs, targeting an approaching threat (Santra and Wüster 2017). Thus, we can speculate that the ventral retinal specialization may be associated with higher spatial resolution of the superior visual field, aiding in the detection of threats ahead of the snake.

Some notable differences in cell density and distribution were observed between the arboreal and ground‐dwelling species analyzed. First, in the highly arboreal colubrids *Oxybelis fulgidus* and *Leptophis ahaetulla*, the mean density of photoreceptors was two to almost three times higher (close to 30,000 cells mm^−^^2^) than in most ground‐dwelling species (ca. 10,000 cells mm^−^^2^). High cone density is likely associated with enhanced visual acuity, which might be crucial for highly arboreal snakes, because vision appears to be a key sensory modality in these species. Among the ground‐dwelling species, the psammophiid *Psammophis elegans* stood out, with a mean density of approximately 17,000 photoreceptors mm^−^^2^. This species is considered highly visual and relies on sight to hunt lizards (Chippaux 1999).

The distribution of SWS1 cones appears to be subtly different between the sampled arboreal and ground‐dwelling caenophidians. In the former, SWS1 cones were relatively scattered throughout the retina, with a higher density in the temporal region. In ground‐dwelling species, SWS1 cones were concentrated in the temporal area in *Naja kaouthia*, in the central retina in *Dryophylax chaquensis* and *Malpolon monspessulanus*, and in the ventral region in the other species. A ventral concentration of SWS1 cones has been reported in other ground‐dwelling (Hauzman et al. 2014; Tashiro et al. 2022) and aquatic snakes (Hauzman et al. 2021). A higher density of short‐wavelength‐sensitive cones in the ventral retina appears to be an adaptation for snakes spending most of their time on the ground, possibly facilitating the detection of dark objects against a brighter sky, as observed in murid rodents (Baden et al. 2013), enhancing the detection of aerial predators.

The distribution of RH1 photoreceptors showed a pronounced difference between the arboreal and ground‐dwelling species, with higher density in the dorsal and ventral retina, respectively. When considering only pure‐RH1 photoreceptors (presumably transmuted, cone‐like rods in the “all‐cone” retinas), their distribution was more restricted to specific areas—dorsal or ventral. These differences in regionalization may be associated with an improved ability to absorb incident light, enhancing vision in low‐light conditions from the lower field (darker forest ground) in arboreal species and from the upper field in ground‐dwelling species.

Finally, variation in the proportions and distribution of SWS1 + RH1 multiopsin cones suggests differences in their importance for the visual functions of each species. In four species, the proportion of multiopsin cones was very low, ranging from 0.1% to 0.4% of the photoreceptors in the arboreal *O. fulgidus* and *L. ahaetulla* and in the ground‐dwelling *N. kaouthia* and *P. elegans*. The areas where they were found were limited to small spots in the retina, either ventral or dorsal. In the other species, the proportion of multiopsin cones accounted for ca. 1%–3% of the photoreceptors. The differences in multiopsin‐cone distribution do not seem to be related to species phylogeny or primary habit (arboreal or ground‐dwelling). It also remains to be seen whether interspecific differences in the regionalization of photoreceptor types exist, or even within an individual's lifespan, given that for most species, only one or two retinas were available for topographic analysis.

Predicting the functional significance of regionalization in photoreceptors is challenging. First, detailed observations of natural history are not available for most snake species, and there have been very few behavioral experiments on the visual ability of any species. Second, the physiology of transmuted cone‐like rods expressing rhodopsin is not yet fully understood, including whether these photoreceptors function under higher light conditions than typical rods and/or contribute to color vision. However, regional differences between multiopsin cones and pure‐SWS1 and pure‐RH1 photoreceptors suggest important functional distinctions among these photoreceptor subtypes in the retinas of caenophidian snakes. Future studies should focus on the functional aspects of transmuted cone‐like rods and multiopsin photoreceptors and the developmental mechanisms underlying photoreceptor differentiation and opsin expression in diurnal and nocturnal snakes. Additionally, it will be particularly valuable to investigate the factors involved in cell‐fate decisions to distinguish photoreceptor classes, as well as the gradients of signaling molecules that allow regional specializations (Rister and Desplan 2011).

## 5 Conclusion

We evaluated aspects of retinal structure and photoreceptor morphology in diurnal and nocturnal caenophidian snakes, using IHC and high‐resolution SEM, and the expression patterns of visual opsins with IHC. Our analyses revealed an extraordinary variability in visual‐cell morphology among species. The use of specific markers for the three visual opsins expressed in snake retinas, SWS1, RH1, and LWS, revealed coexpression of SWS1 and LWS opsins in some cones of nocturnal species (with duplex retinas). In the “all‐cone” retinas of diurnal species, the rod opsin (RH1) was not only expressed purely in transmuted, cone‐like rods (that have superficially cone‐like outer segments but retain rod‐like synaptic terminals) but was also widely coexpressed with the SWS1 cone opsin in (apparently primarily SWS1) cone photoreceptors, an unprecedented finding for vertebrates.

In his comprehensive book on the comparative anatomy of the vertebrate eye and retina, the visual anatomist Gordon L. Walls (1942) stated that “*snakes alone have rung as many changes upon their visual‐cell patterns as have all the other vertebrates put together*.” More than eight decades later, we continue to discover additional ways in which snake retinas display exceptional diversity and adaptive innovations. The unique features and striking diversity of their visual cells, even among closely related species, highlight snakes as an outstanding group for studying the function and evolution of vertebrate visual systems.

## Author Contribution

E.H., S.H., and D.J.G. conceived and designed the study, wrote the first draft, and revised the reviewed manuscript. J.H.T., I.L.G., N.F.T.V., T.B.G., P.N., N.R.C., M.E.O., A.L.C.P., D.O.M., and D.F.V. contributed to pre‐submission manuscript revisions. E.H., S.H., D.F.V., and D.J.G. obtained funding. N.F.T.V., T.B.G., P.N., N.R.C., C.M.M., M.E.O., A.L.C.P., and D.O.M. collected snakes, provided retinal samples, and/or advised on snake ecology. E.H., S.H., J.H.T., I.L.G., and D.J.G. prepared, imaged, analyzed, and/or interpreted retinas. All authors read and approved the final manuscript.

## Ethics Statement

The permit for specimen collection was issued by the Brazilian Ministry of the Environment and the competent authority, the Chico Mendes Institute for Biodiversity Conservation (SISBIO 79155, SISBIO 86246). All procedures were in accordance with ethical principles of animal management and experimentation established by the Brazilian Council for Control of Animal Experimentation (CONCEA) and approved by the Ethics Committee of Animal Research of the Psychology Institute, University of São Paulo, Brazil (permission number 9284040521); the Institutional Animal Care and Use Committee at Charles University in Prague and the Ministry of Culture of the Czech Republic (permission number UKPRF/28830/2021); or the UK Home Office and the LSTM Animal Welfare and Ethical Review Board (establishment license X20A6D134).

## Conflicts of Interest

The authors declare no conflicts of interest.

## Peer Review

The peer review history for this article is available at https://publons.com/publon/10.1002/cne.70092.

## Supporting information



Supplementary Table S1. Snake specimens collected and voucher numbers.
Supplementary Table S2. Caenophidian snakes analyzed, their photoreceptor complements, and visual opsins expressed. Vernacular names for species are from Reptile Database (Uetz et al. 2025) except those marked with asterisk (*), which are proposed here based on etymology of scientific names and/or vernacular names of close relatives. Vernacular names for families from Gower et al. (2023). Names of photoreceptors in parentheses are following the nomenclature of Baden et al. (2025). Dashes (–) indicate absence of a particular type of photoreceptor and/or visual‐opsin expression; question mark indicates that insuficient material was examined to be confident about a potential absence and/or retinas were labeled with antibodies against only SWS1 and RH1, and therefore, possible coexpression in cones with LWS is unknown. For species indicated with two asterisks (**), only one or two retinas were available and used only for wholemounts (not sections). Nocturnal taxa shaded grey.
Supplementary Table S3. Stereological parameters used to estimate the number of photoreceptors.
Supplementary Figure S1. Retinal sections of nine nocturnal and nine diurnal endoglyptodontan caenophidian snakes, showing the nuclear layers labeled with DAPI (gray). ONL, outer nuclear layer; INL, inner nuclear layer; GCL, ganglion cell layer. The exact retinal region of each section is unknown. Scale bars: 20 µm.
Supplementary Figure S2. Retinal cross‐sections of the nocturnal dipsadid snake *D. chaquensis*, showing a diurnal retinal pattern. The nuclear layers are labeled with DAPI (blue). ONL, outer nuclear layer; INL, inner nuclear layer; GCL, ganglion cell layer.
Supplementary Figure S3. Topographic maps of the retinas of caenophidian snakes.

## Data Availability

The data that support the findings of this study are available from the corresponding author upon reasonable request.
