# Supplementary material for: Exceptional Visual‐Opsin Coexpression and Phenotypic Diversity in Outer‐Retinal Photoreceptors of Caenophidian Snakes
Source: J Comp Neurol. 2025 Oct 7;533(10):e70092. doi: 10.1002/cne.70092 (PMC12501914; doi:10.1002/cne.70092)
Supplement: Supplementary file 1 — Supplementary Table S1. Snake specimens collected and voucher numbers. Supplementary Table S2. Caenophidian snakes analyzed, their photoreceptor complements, and visual opsins expressed. Vernacular names for species are from Reptile Database (Uetz et al. 2025) except those marked with asterisk (*), which are proposed here based on etymology of scientific names and/or vernacular names of close relatives. Vernacular names for families from Gower et al. (2023). Names of photoreceptors in parentheses are following the nomenclature of Baden et al. (2025). Dashes (–) indicate absence of a particular type of photoreceptor and/or visual‐opsin expression; question mark indicates that insuficient material was examined to be confident about a potential absence and/or retinas were labeled with antibodies against only SWS1 and RH1, and therefore, possible coexpression in cones with LWS is unknown. For species indicated with two asterisks (**), only one or two retinas were available and used only for wholemounts (not sections). Nocturnal taxa shaded grey. Supplementary Table S3. Stereological parameters used to estimate the number of photoreceptors. Supplementary Figure S1. Retinal sections of nine nocturnal and nine diurnal endoglyptodontan caenophidian snakes, showing the nuclear layers labeled with DAPI (gray). ONL, outer nuclear layer; INL, inner nuclear layer; GCL, ganglion cell layer. The exact retinal region of each section is unknown. Scale bars: 20 µm. Supplementary Figure S2. Retinal cross‐sections of the nocturnal dipsadid snake D. chaquensis, showing a diurnal retinal pattern. The nuclear layers are labeled with DAPI (blue). ONL, outer nuclear layer; INL, inner nuclear layer; GCL, ganglion cell layer. Supplementary Figure S3. Topographic maps of the retinas of caenophidian snakes. [file CNE-533-e70092-s001.pdf]

Exceptional visual-opsin coexpression and phenotypic diversity in outer-retinal photoreceptors of caenophidian snakes.

Einat Hauzman, Silke Haverkamp, Juliana H. Tashiro, Irene Gügel, Natalia F. Torello-Viera, Thaís B. Guedes, Pavel Němec, Nicholas R. Casewell, Cassandra M. Modahl, Maria Ermelinda Oliveira, Ana Lúcia C. Prudente, Daniel O. Mesquita, Dora Fix Ventura, David J. Gower

Supporting Information

Supplementary Table S1. Snake specimens collected and voucher numbers.

Family	Species	ID	Location	Sex	Size	Deposit Location/ Voucher
Viperidae	*Bothrops jararaca*	Bjar230112.1*	São Paulo, SP	F	SVL+TL=425+48 mm; m=22.3g	-
	Bothrops jararacussu	Bjus230112.1*	São Paulo, SP	M	SVL+TL=303+35.5 mm; m=16.4g	-
Psammophiidae	*Psammophis elegans*	PEL1	Prague, Czechia	M	SVL+TL=1000+600 mm; m=119g	DZCHU-PEL1
	Malpolon monspessulanus	Malp1				NHM 2022.07576
Elapidae	*Naja kaouthia*	NajKao.CB003	Captive bred Liverpool, UK	F		-
	Naja haje	NajNivZAF004	Lierpool, UK			-
Colubridae	*Chironius flavolineatus*	ITV699	São Geraldo do Araguaia, PA	F	SVL+TL=66.3+33.5 mm; m=70g	MPEG 27351
	Leptophis ahaetulla	Lahe220118.1	Manaus, AM	M	SVL+TL=635+41 mm	UFAM – CZPB-RP-1072
		Lahe220118.2	Manaus, AM	M	SVL+TL=520+326 mm	UFAM – CZPB-RP-1073
	Oxybelis fulgidus	Oful220118	Manaus, AM	F	SVL+TL=1025+510 mm	UFAM – CZPB-RP-1074
	Mastigodryas boddaerti	Mbod240328	Chapada dos Guimarães, MS	F	SVL+TL=860+310 mm	UFJF – MAP 11238
	Pantherophis guttatus	EGU1	Prague, Czechia	M	SVL+TL=1208+208 mm; m=584g	DZCHU-EGU1
Natricidae	*Thamnophis sirtalis*	TSI1	Prague, Czechia	F	SVL+TL=543+172 mm; m=50g	DZCHU-TSI1
Dipsadidae	*Dryophylax phoenix*	Tpho-12736	Campo Formoso, BA	F	SVL+TL=516+83.8 mm; m=41g	FSCH-UFPB 12736
	Dryophylax chaquensis	Tchaq-221212	Poconé, MT	F	SVL+TL=442+116 mm; m=33.2 g	UFMT-R 12.808
		Tchaq-191125	Porto Murtinho, MS	F	SVL+TL=444+115 mm; m=33 g	IBSP091913
		Tchaq-220505*	Poconé, MT	M	SVL+TL=265+66 mm; m=8 g	UFMT-R 12.710
		Tchaq-16	Serra do Amolar, MT	M	SVL+TL=461+146 mm; m=51 g	UFMT-R 12.826
		Tchaq-33	Poconé, MT	M	SVL+TL=432+132 mm; m=29.2 g	UFMT-R 12.978
	Tomodon dorsatus	Tdor211112	Cotia, SP	M	SVL+TL=392+125 mm; m=23.9 g	IBSP95402
		Tdor211125	P.E. Cantareira, SP		SVL+TL=600+200 mm; m=67.4 g	IBSP95375
		Tdor10-220602	São Paulo, SP	F	SVL+TL=400+93 mm; m=35.4 g	IBSP95405
		Tdor190930	São Roque, SP	F		IBSP091727

	Philodryas patagoniensis	Ppat230203*	São Paulo, SP	M	SVL+TL=350+140 mm; m=14 g	IBSP95378
	Chlorosoma viridissimus	Cvir220117*	Manaus, AM	F	SVL+TL=342+120 mm	UFAM – CZPB-RP-1071
	Dipsas mikanii	Smik221216	São Paulo, SP	F	SVL+TL=430+75 mm; m=26.7g	IBSP95376
	Dipsas neuwiedi	Sneu221216	São Paulo, SP	F	SVL+TL=480+125 mm	IBSP95377
	Oxyrhopus guibei	Oguib221216*	São Paulo, SP		SVL+TL=200+50 mm; m=5.2 g	IBSP95379
	Oxyrhopus trigeminus	Otrig-12357	Campo Formoso, BA	F	SVL+TL=355+67 mm; m=13,5g	FSCH-UFPB 12357
	Pseudoboa nigra	Pnig-12277	Campo Formoso, BA	M	SVL+TL=824+227 mm; m=210g	FSCH-UFPB 12277
	Leptodeira annulata	ITV549	São Geraldo do Araguaia, PA	F	SVL+TL=42.5+12.8 mm; m=30g	MPEG 27353
	Leptodeira tarairiu	Ltar-12797	Campo Formoso, BA	M	SVL+TL=520+187 mm; m=28,5g	FSCH-UFPB 12797

*juvenile specimens; SVL, snout vent length; TL, tail length; m, mass; DZCHU, Department of Zoology, Charles University, Prague; MPEG, Museu Paraense Emílio Goeldi; NHM, Natural History Museum, London; UFAM, Universidade Federal do Amazonas; UFMT, Universidade Federal de Mato Grosso; UFMS, Universidade Federal do Mato Grosso do Sul; UFPB, Universidade Federal da Paraíba. UFJF, Universidade Federal de Juiz de Fora.

Supplementary Table S2. Caenophidian snakes analyzed, their photoreceptor complements, and visual opsins expressed. Vernacular names for species are from Reptile Database (Uetz et al. 2025) except those marked with asterisk (*), which are proposed here based on etymology of scientific names and/or vernacular names of close relatives. Vernacular names for families from Gower et al. (2023). Names of photoreceptors in parentheses are following the nomenclature of Baden et al. (2025). Dashes (–) indicate absence of a particular type of photoreceptor and/or visual-opsin expression; question mark indicates that insuficient material was examined to be confident about a potential absence and/or retinas were labeled with antibodies against only SWS1 and RH1, and therefore, possible coexpression in cones with LWS is unknown. For species indicated with two asterisks (**), only one or two retinas were available and used only for wholemounts (not sections). Nocturnal taxa shaded grey.

Family	Species	Vernacular name	Diel activity	Retinal type	Photoreceptors and visual opsins they express							
					Typical rods (PR0)	Transmuted cone-like rods (PR0)	Small single cones (PR4)				Large single cones (PR1)	Double cones
COLUBRIDAE Rat Snakes, Tree Snakes, Racers, King Snakes, and relatives	*Chironius flavolineatus***	Boettger's Sipo	Diurnal	“All-cone”	–	RH1	SWS1	SWS1+RH1	?	?	LWS	LWS
	Leptophis ahaetulla	(Giant) Parrot Snake	Diurnal	“All-cone”	–	RH1	SWS1	SWS1+RH1	SWS1+LWS	?	LWS	LWS
	*Mastigodryas boddaerti***	Boddaert's Tropical Racer	Diurnal	“All-cone”	–	RH1	SWS1	SWS1+RH1	?	?	LWS	LWS
	*Oxybelis fulgidus***	Green Vine Snake	Diurnal	“All-cone”	–	RH1	SWS1	SWS1+RH1	?	?	LWS	LWS
	Pantherophis guttatus	Red Cornsnake	Diurnal	“All-cone”	–	RH1	SWS1	SWS1+RH1	?	?	LWS	LWS
DIPSADIDAE New World Snail-eating Snakes, Cat-eyed Snakes, Mussuranas, False Pit Vipers, and relatives	*Chlorosoma viridissimum*	Common Green Racer	Diurnal	“All-cone”	–	RH1	SWS1	SWS1+RH1	?	?	LWS	LWS
	Dipsas mikanii	Mikan's Tree Snake*	Nocturnal	Duplex	RH1	–	SWS1	–	SWS1+LWS	–	LWS	–
	Dipsas neuwiedi	Neuwied's Tree Snake	Nocturnal	Duplex	RH1	–	SWS1	–	SWS1+LWS	–	LWS	–
	*Dryophylax chaquensis***	False Jararaca	Nocturnal	“All-cone”	–	RH1	SWS1	SWS1+RH1	?	?	LWS	LWS
	*Dryophylax phoenix***	Sword Snake*	Nocturnal	“All-cone”	–	RH1	SWS1	SWS1+RH1	?	?	LWS	LWS
	Leptodeira annulata	Banded Cat-eyed Snake	Nocturnal	Duplex	RH1	–	SWS1	–	SWS1+LWS	–	LWS	LWS
	Leptodeira tarairiu	Tarairiú Cat-eyed Snake*	Nocturnal	Duplex	RH1	–	SWS1	–	SWS1+LWS	–	LWS	LWS
	Oxyrhopus guibei	Guibé's False Coral Snake*	Nocturnal	Duplex	RH1	–	SWS1	–	SWS1+LWS	–	LWS	LWS
	Oxyrhopus trigeminus	Brazilian False Coral Snake	Nocturnal	Duplex	RH1	–	SWS1	–	SWS1+LWS	–	LWS	LWS
	Philodryas patagoniensis	Patagonian Green racer	Diurnal	“All-cone”	–	RH1	SWS1	SWS1+RH1	?	?	LWS	LWS
	Pseudoboa nigra	Black False Boa	Nocturnal	Duplex	RH1	–	SWS1	–	SWS1+LWS	–	LWS	LWS
	Tomodon dorsatus	Pampas Snake	Diurnal	“All-cone”	–	RH1	SWS1	SWS1+RH1	SWS1+LWS	SWS1+RH1 +LWS	LWS	LWS
ELAPIDAE Cobras, Coral Snakes, Kraits, Taipans and Sea Snakes	*Naja haje*	Egyptian Cobra	Diurnal	“All-cone”	–	RH1	SWS1	SWS1+RH1	SWS1+LWS	?	LWS	LWS
	*Naja kaouthia***	Monocled Cobra	Diurnal	“All-cone”	–	RH1	SWS1	SWS1+RH1	?	?	LWS	LWS
NATRICIDAE Grass Snakes, Marsh Snakes, Keelbacks, Garter Snakes and Water Snakes	*Thamnophis sirtalis*	Common Garter Snake	Diurnal	“All-cone”	–	RH1	SWS1	?	?	SWS1+RH1 +LWS	LWS	LWS
PSAMMOPHIIDAE Sand Snakes, Skaapstekers, Bark Snakes and Beaked Snakes	*Malpolon monspesulanus*	Montpellier Snake	Diurnal	“All-cone”	–	RH1	SWS1	SWS1+RH1	SWS1+LWS	?	LWS	LWS
	Psammophis elegans	Elegant Sand Racer	Diurnal	“All-cone”	–	RH1	SWS1	SWS1+RH1	?	SWS1+RH1 +LWS	LWS	LWS
VIPERIDAE Vipers	*Bothrops jararaca*	Jararaca, lancehead	Nocturnal	Duplex	RH1	–	SWS1	–	–	–	LWS	LWS
	Bothrops jararacussu	Jararacussu	Nocturnal	Duplex	RH1	–	SWS1	–	–	–	LWS	LWS

Supplementary Table S3. Stereological parameters used to estimate the number and distribution of photoreceptors in retinas of caenophidian snakes, using the optical fractionator method.

Species	Retina (ID)	Retinal Area (mm^2)	Counting Frame (µm x µm)	Grid (µm x µm)	Area of sampling fraction (asf)	Number of sites counted
Chironius flavolineatus	Cfla1-RE	29.9	100 x 100	400 x 400	0.056	166
Leptophis ahaetulla	Laha1-RE	23	100 x 100	300 x 300	0.1	230
	Laha2-LE	23.6	100 x 100	350 x 350	0.075	178
Oxybelis fulgidus	Oful1-RE	36.1	100 x 100	400 x 400	0.06	215
	Oful1-LE	36	100 x 100	400 x 400	0.06	210
Mastigodryas boddaerti	Mbod1-LE	50.6	100 x 100	450 x 450	0.045	260
	Mbod1-RE	48.5	100 x 100	450 x 450	0.042	254
Psammophis elegans	Pel1-	40.6	100 x 100	400 x 400	0.05	201
Malpolon monspessulanus	Malp1-RE	87.8	100 x 100	600 x 600	0.02	181
Naja kaouthia	Nkao1	65	100 x 100	500 x 500	0.03	196
Dryophylax phoenix	Tpho1-RE	23.3	100 x 100	300 x 300	0.11	254
Dryophylax chaquensis	Tchaq1-LE	15.6	100 x 100	250 x 250	0.14	216
	Tchaq2-LE	29.1	100 x 100	350 x 350	0.073	212
	Tchaq3-RE	11.2	100 x 100	250 x 250	0.13	143
	Tchaq4-LE	22.2	100 x 100	350 x 350	0.068	150
Tomodon dorsatus	Tdor1-RE	22.3	100 x 100	350 x 350	0.077	171
	Tdor1-LE	22	100 x 100	350 x 350	0.078	171
	Tdor2-RE	30.2	100 x 100	350 x 350	0.065	196
	Tdor2-LE	30.4	100 x 100	400 x 400	0.055	168
	Tdor3-LE	17.5	100 x 100	300 x 300	0.103	181
	Tdor4-RE	24.3	100 x 100	350 x 350	0.075	183

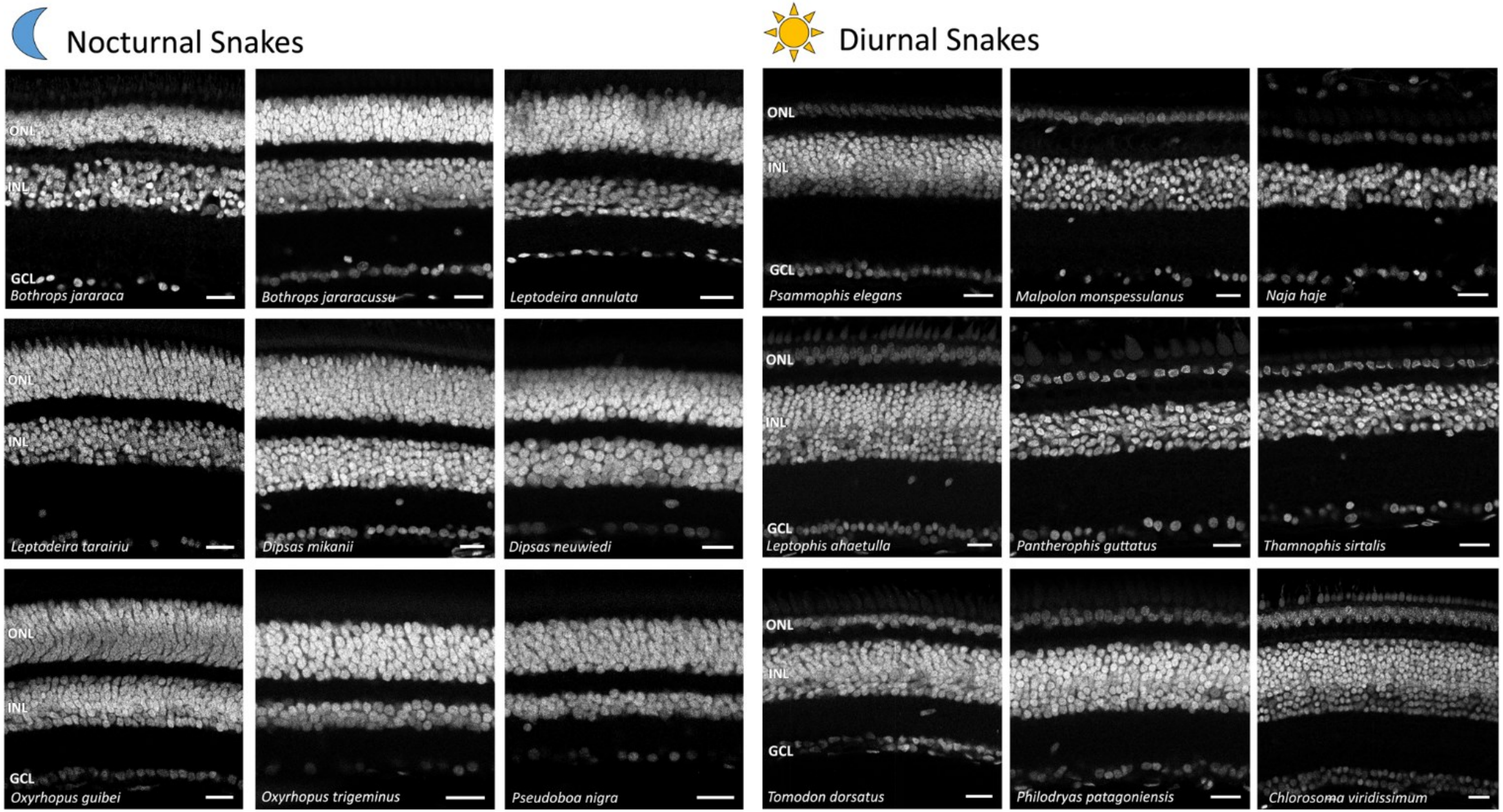

Supplementary Figure S1. Retinal sections of nine nocturnal and nine diurnal endoglyptodontan caenophidian snakes, showing the nuclear layers labeled with DAPI (gray). ONL, outer nuclear layer; INL, inner nuclear layer; GCL, ganglion cell layer. The exact retinal region of each section is unknown. Scale bars: 20 µm.

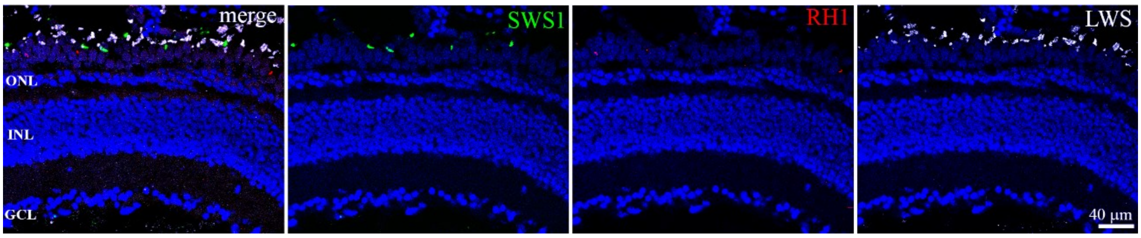

Supplementary Figure S2. Retinal cross-sections of the 'nocturnal' dipsadid snake *Dryophylax chaquensis*, showing a diurnal retinal pattern. The nuclear layers are labeled with DAPI (blue). ONL, outer nuclear layer; INL, inner nuclear layer; GCL, ganglion cell layer.

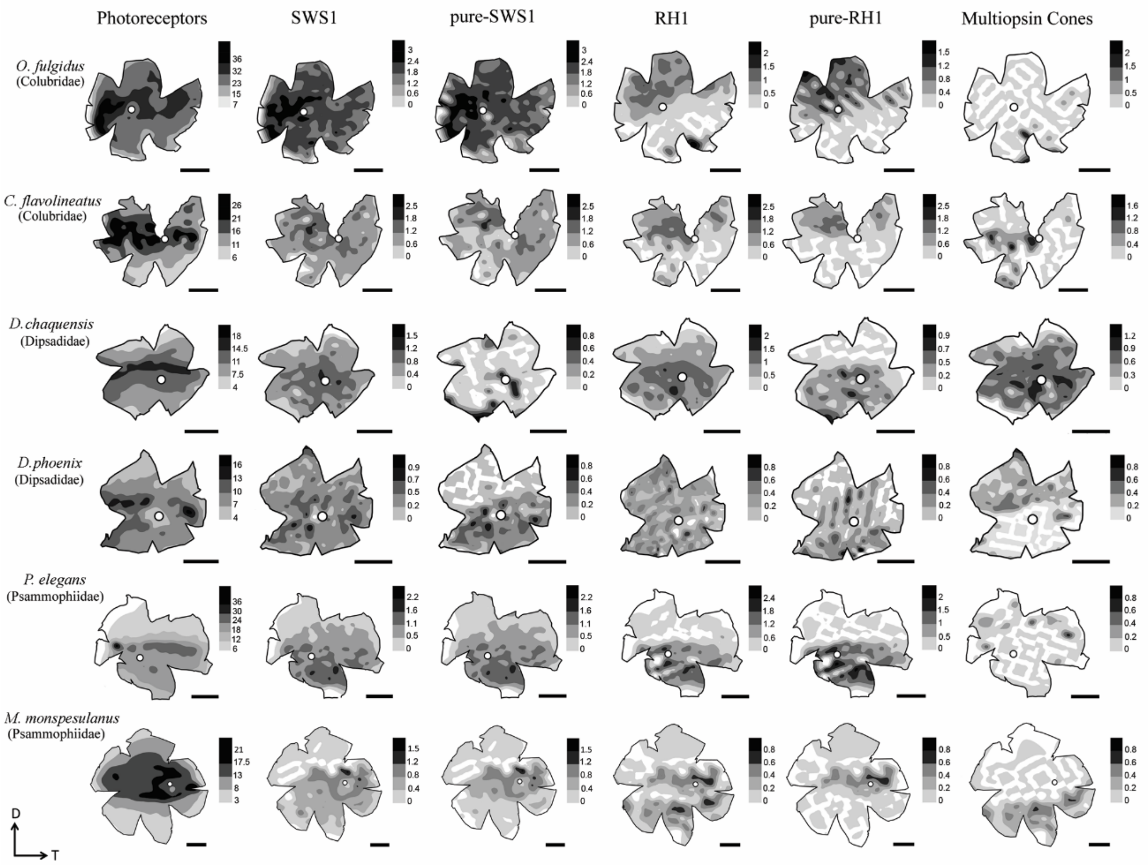

Supplementary Figure S3. Topographic maps of the retinas of caenophidian snakes: the arboreal colubrids *Oxybelis fulgidus* and *Chironius flavolineatus*, and the ground-dwelling dipsadids *Dryophylax chaquensis* and *D. phoenix*, and psammophiids *Psammophis elegans* and *Malpolon monspessulanus*, showing different distribution patterns of i) total photoreceptors, ii) SWS1-expressing cones (pure-SWS1 and SWS1+RH1 multiopsin cones), iii) pure-SWS1 cones, iv) RH1-expressing photoreceptors (pure-RH1 cone-like rods and SWS1+RH1 multiopsin cones), v) pure-RH1 cone-like rods, and vi) SWS1+RH1 multiopsin cones. Gray shaded bars indicate the number of cells mm^{-2}. The values should be multiplied by 10^3. The optic nerve head is depicted as a white circle. D, dorsal; T, temporal.

References:

Baden, T., Angueyra, J. M., Bosten, J. M., Collin, S. P., Conway, B., Cortesi, F., Dedek, K., Euler, T., Flamarique, I. N., Franklin, A., Haverkamp, S., Kelber, A., Neuhauss, S. C. F., Li, W., Lucas, R., Osorio, D. C., Shekhar, K., Tommasini, D., Yoshimatsu, T., and Corbo, J. C. 2025. A standardized nomenclature for the rods and cones of the vertebrate retina. *PLoS Biology*, 23(5): e3003157.

Gower, D.J., Garrett, K. & Maddock, S.T. (2023) *Snakes. Their diversity, ecology and behaviour*. Natural History Museum, London. 192 pp.

Uetz, P., Freed, P, Aguilar, R., Reyes, F., Kudera, J. & Hošek, J. (eds.). 2025. The Reptile Database, http://www.reptile-database.org, accessed 08 August 2025.
